# Self-organizing three-dimensional dermal papilla cell spheroids yield therapeutic extracellular vesicles that target hypertrophic scar regression via the miR-26a-5p/CCNE2 axis

**DOI:** 10.1093/burnst/tkaf048

**Published:** 2025-07-22

**Authors:** Yunwei Wang, Luyang Zhao, Hao Ma, Ao Shi, Peng Cao, Feiyu Cai, Ruomei Zhao, Gang Wang, Zhihan Hu, Jiatong Wang, Yuchen Kang, Xiaoyu Di, Qingyi Zhang, Hao Zhang, Shuguang Hou, Babo Zhang, Liang Luo, Han Wang, Yi Liu, Hao Guan

**Affiliations:** Department of Burns and Cutaneous Surgery, Xijing Hospital, Fourth Military Medical University, 127 West Changle Road, Xi’an, Shaanxi 710032, China; Department of Burn Plastic and Wound Repair Surgery, Lanzhou University Second Hospital, No. 82 Cuiyingmen, Lanzhou, Gansu 730030, China; Department of Burns and Cutaneous Surgery, Xijing Hospital, Fourth Military Medical University, 127 West Changle Road, Xi’an, Shaanxi 710032, China; Department of Plastic and Reconstructive Surgery, Shanghai Ninth People’s Hospital affiliated to Shanghai Jiao Tong University School of Medicine, No. 639 Zhizaoju Road, Huangpu District, Shanghai 200011, China; Department of Burn Plastic and Wound Repair Surgery, Lanzhou University Second Hospital, No. 82 Cuiyingmen, Lanzhou, Gansu 730030, China; Burns & Trauma Treatment Center, Affiliated Hospital of Jiangnan University, No. 1000 Hefeng Road, Binhu District, Wuxi, Jiangsu 214122, China; Department of Burn Plastic and Wound Repair Surgery, Lanzhou University Second Hospital, No. 82 Cuiyingmen, Lanzhou, Gansu 730030, China; Department of Burn Plastic and Wound Repair Surgery, Lanzhou University Second Hospital, No. 82 Cuiyingmen, Lanzhou, Gansu 730030, China; Department of Burn Plastic and Wound Repair Surgery, Lanzhou University Second Hospital, No. 82 Cuiyingmen, Lanzhou, Gansu 730030, China; Department of Burn Plastic and Wound Repair Surgery, Lanzhou University Second Hospital, No. 82 Cuiyingmen, Lanzhou, Gansu 730030, China; Department of Burn Plastic and Wound Repair Surgery, Lanzhou University Second Hospital, No. 82 Cuiyingmen, Lanzhou, Gansu 730030, China; Department of Burn Plastic and Wound Repair Surgery, Lanzhou University Second Hospital, No. 82 Cuiyingmen, Lanzhou, Gansu 730030, China; Department of Burn Plastic and Wound Repair Surgery, Lanzhou University Second Hospital, No. 82 Cuiyingmen, Lanzhou, Gansu 730030, China; Department of Burns and Cutaneous Surgery, Xijing Hospital, Fourth Military Medical University, 127 West Changle Road, Xi’an, Shaanxi 710032, China; Department of Burns and Cutaneous Surgery, Xijing Hospital, Fourth Military Medical University, 127 West Changle Road, Xi’an, Shaanxi 710032, China; Department of Burns and Cutaneous Surgery, Xijing Hospital, Fourth Military Medical University, 127 West Changle Road, Xi’an, Shaanxi 710032, China; Department of Burns and Cutaneous Surgery, Xijing Hospital, Fourth Military Medical University, 127 West Changle Road, Xi’an, Shaanxi 710032, China; Department of Burns and Cutaneous Surgery, Xijing Hospital, Fourth Military Medical University, 127 West Changle Road, Xi’an, Shaanxi 710032, China; Department of Orthopedics, Air Force Medical Center, No. 30, Fucheng Road, Beijing 100142, China; Department of Burn Plastic and Wound Repair Surgery, Lanzhou University Second Hospital, No. 82 Cuiyingmen, Lanzhou, Gansu 730030, China; Department of Burns and Cutaneous Surgery, Xijing Hospital, Fourth Military Medical University, 127 West Changle Road, Xi’an, Shaanxi 710032, China

**Keywords:** Hypertrophic scar, Extracellular vesicles, Three-dimensional spheroids, Dermal papilla cells, CCNE2, microRNA-26a-5p

## Abstract

**Background:**

Hypertrophic scarring remains a critical challenge in regenerative medicine because of the limited efficacy of current antifibrotic therapies. Although dermal papilla cells (DPCs) exhibit intrinsic scar-inhibitory potential, their therapeutic utility is constrained by rapid replicative senescence and poor scalability in traditional monolayer cultures, necessitating innovative strategies to enhance cellular functionality and manufacturing feasibility.

**Methods:**

A self-feeder layer 3D (SFL-3D) platform was established to reprogram primary human DPCs into rejuvenated three-dimensional DPC (tdDPC) spheroids via autocrine–paracrine signalling activation. tdDPC-derived extracellular vesicles (tdDPC-EVs) were isolated from culture supernatants by differential centrifugation. The antifibrotic effects of tdDPC-EVs were systematically evaluated using human scar fibroblasts through scratch wound healing assays, CCK-8 proliferation assays, and fibrotic marker analysis [Western blotting and immunofluorescence staining for α-smooth muscle actin (α-SMA) and collagen I]. Bioinformatics was used to predict key pathways involved in hypertrophic scar (HS) pathogenesis, whereas gain/loss-of-function studies investigated the miR-26a-5p/CCNE2 regulatory axis. Therapeutic validation was performed in a rabbit ear hypertrophic scar model with histopathological and molecular profiling.

**Results:**

Compared with conventional 3D cultures, the SFL-3D system demonstrated superior proliferative support, enabling stable tdDPC expansion beyond 10 passages while maintaining high viability and enhanced EV biogenesis. miR-26a-5p-enriched tdDPC-EVs attenuated fibrosis through two mechanisms: (1) silencing CCNE2 to block PI3K/AKT-driven collagen overproduction and (2) suppressing α-SMA + myofibroblast differentiation. In the rabbit ear HS model, tdDPC-EV administration reduced the scar elevation index and restored the collagen I/III ratio to near-physiological levels.

**Conclusions:**

This study positions tdDPC-EVs as a scalable acellular therapy that overcomes the replicative senescence and manufacturing limitations of cellular approaches. The antiscarring efficacy of these EVs, which is mediated by the miR-26a-5p/CCNE2/PI3K/AKT axis, highlights their clinical potential as precision-targeted strategies for hypertrophic scar management. The SFL-3D platform further provides a translatable framework for EV-based regenerative therapeutics.

## Graphical Abstract

**Graphical Abstract:**
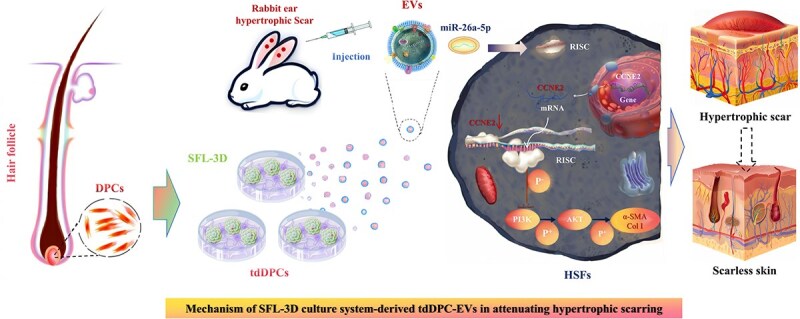

## Highlights

- A SFL-3D enables scalable production of rejuvenated dermal papilla cell spheroids (tdDPCs) with enhanced proliferative capacity and EV biogenesis.
- tdDPC-EVs effectively attenuate hypertrophic scar formation by suppressing fibroblast proliferation, migration, and myofibroblast differentiation.
- Mechanistically, tdDPC-EVs deliver miR-26a-5p to target CCNE2, thereby inhibiting the PI3K/AKT signaling pathway and disrupting fibrotic progression both in vitro and in vivo.
- This study establishes a novel cell-free therapeutic strategy using engineered EVs for hypertrophic scar management, overcoming limitations of cellular therapies and conventional 3D culture systems.

## Background

Hypertrophic scarring (HS) remains a devastating sequela of burn injuries and surgical trauma, with epidemiological data indicating that it affects 40%–70% of burn survivors and up to 91% of surgical patients worldwide [1]. These disfiguring fibrotic lesions impose lifelong physical and psychological burdens, whereas current therapies, such as corticosteroid injections and laser ablation, provide only transient symptomatic relief [2, 3]. Given these challenges, the development of novel therapeutic strategies capable of addressing pathological fibrosis at its source is urgently needed.

Emerging evidence highlights hair follicle–derived mesenchymal cells as potent modulators of dermal fibrosis. Importantly, dermal papilla cells (DPCs)—architectural organizers of folliculogenesis—exhibit a unique capacity to suppress pathological fibroblast activation through paracrine signalling. However, conventional 2D expansion induces premature senescence, with EV production decreasing by >80% by passage 5 [4]. Preclinical studies have revealed that transplanted DPCs significantly improve scar pliability and the collagen architecture through the paracrine modulation of fibroblast behaviour [4, 5]. However, the clinical translation of cellular therapies faces significant barriers, including immunogenicity risks, cryopreservation challenges, and potential ectopic differentiation.

In contrast, extracellular vesicles (EVs) derived from DPCs have recently garnered attention as a cell-free alternative. Notably, these nanoscale particles inherit the therapeutic cargo of the parental cells while circumventing the critical limitations of cellular transplants; e.g. their lipid bilayer structure confers immune privilege, enables lyophilized storage without a loss of potency, and permits systemic delivery across biological barriers [6]. Moreover, comparative studies have confirmed that DPC-EVs retain >80% of the antifibrotic efficacy of cellular transplants while eliminating the risks associated with cell survival and differentiation [7]. Collectively, these advantages position EVs as a scalable and minimally invasive strategy for scar modulation.

Despite these advantages, industrial-scale EV production remains constrained by donor cell senescence in conventional culture systems. Two-dimensional (2D)-cultured DPCs rapidly lose functional potency, with EV yields and miRNA cargo quality deteriorating over serial passages [4]. Three-dimensional (3D) culture innovations partially mitigate cellular dedifferentiation but introduce new bottlenecks. Scaffold-free approaches using ultralow attachment surfaces often generate irregular spheroids prone to fusion and hypoxic necrosis, whereas bioreactor systems struggle to maintain the consistency of EV biogenesis across batches [8, 9]. These technical hurdles perpetuate a critical translational gap: scaling EV production necessitates extended cell passaging, which paradoxically erodes therapeutic potency through senescence-associated cargo alterations.

We bridged this gap by developing a self-feeder layer 3D (SFL-3D) culture system that harnesses cellular heterogeneity to sustain long-term EV production. In this spontaneously organized architecture, proliferative DPC subpopulations form semiadherent spheroids atop a self-renewing feeder layer, mimicking the dynamic equilibrium of native hair follicle niches. The adherent layer not only provides structural support through endogenous extracellular matrix deposition but also secretes growth factors that maintain spheroid viability during extended cultures. Unlike forced aggregation methods, this design prevents spheroid fusion and hypoxia-induced apoptosis, enabling stable EV harvesting with enriched antifibrotic miRNAs. This method produces DPC spheroids, termed three-dimensional DPCs (tdDPCs), which maintain high proliferation and vesicle secretion capabilities for at least 10 passages, laying the foundation for the next step of extracting EVs and studying their mechanisms of alleviating HS.

In this study, we demonstrated that tdDPC-derived EVs (tdDPC-EVs) effectively alleviated HS by the dual modulation of pathological fibrosis and cellular hyperactivation. *In vitro* analyses indicated that tdDPC-EVs significantly suppressed the pathological activation of hypertrophic scar-derived fibroblasts (HSFs), effectively reducing fibrotic marker expression and restraining myofibroblast differentiation. *In vivo* evaluations using rabbit ear models showed improved wound healing quality with a substantial reduction in HS characteristics, particularly collagen overassembly and persistent myofibroblast activation. Mechanistically, tdDPC-EVs leverage their miR-26a-5p payload to silence cyclin E2 (CCNE2), a master cell cycle coordinator whose pathological overexpression drives HS progression through convergent biological programmes. This dual regulatory capacity enables tdDPC-EVs to disrupt the vicious cycle in which CCNE2-sustained fibroblast proliferation reinforces matrix stiffening, which in turn exacerbates cellular mechanoactivation. Notably, tdDPC-EVs achieve the coordinated blockade of these processes via the selective enrichment of miR-26a-5p, which silences CCNE2 to concurrently normalize the fibroblast cycle and disrupt AKT-mediated fibrotic cascades. Collectively, our findings establish the SFL-3D-cultured EV platform as a novel therapeutic strategy that effectively disrupts the pathognomonic vicious cycle between cellular hyperactivity and maladaptive matrix remodelling in HS. Moreover, our study shows for the first time the therapeutic application of SFL-3D-cultured tdDPC-EVs in hypertrophic scar regression via the newly identified miR-26a-5p/CCNE2 axis. Our findings complement previous work on the proangiogenic effects of DPC-EVs by addressing the antifibrotic potential of SFL-3D-derived EVs. These findings establish the foundation for DPC-derived EV-targeted therapy in HS, providing a dual-axis strategy to disrupt the self-perpetuating fibrosis cycle.

## Methods

### Patients and ethical approval

HS specimens and follicular units were obtained from patients (mean age: 30.0 ± 4.2 years) undergoing scar revision surgery at Lanzhou University Second Hospital. Written informed consent was secured following the full disclosure of the research objectives and procedures. All protocols were approved by the Ethics Committee of Lanzhou University Second Hospital (Approval No. 2024A-558) in compliance with the principles of the Declaration of Helsinki.

### DPC isolation and SFL-3D culture

Primary DPCs were carefully isolated from human follicular units. End bulbs were cultured in high-glucose Dulbecco’s modified Eagle’s medium (DMEM) supplemented with 20% foetal bovine serum (FBS; Biological Industries) in a sterile Petri dish. Primary DPCs were maintained in high-glucose DMEM supplemented with 10% FBS for passages 1–5 with 5% CO_2_ at 37°C. After five passages, the cells were switched to DMEM/F-12 supplemented with 10% FBS.

After primary DPCs were dissociated for 5 min with StemPro™ Accutase™ Reagent (Gibco), a single-cell suspension was obtained. Based on previous reports, 5 × 10^4^ cells/cm^2^ in self-feeder layer 3D (SFL-3D) culture medium (containing 2% FBS, 5 ng/ml human bFGF, 2 ng/ml human EGF, 5 ng/ml human PDGF, 2 μg/ml heparin, 50 μg/ml L-ascorbic acid 2-phosphate sesquimagnesium salt hydrate, 100 U/ml penicillin, and 100 μg/ml streptomycin) were prepared for the cell suspension [4]. DPCs cultured under these three-dimensional conditions are referred to as tdDPCs.

### Sphere vitality and functional characterization of the SFL-3D and ULA-3D DPCs

A spheroid viability assessment was performed under standardized conditions. For ultralow adhesion 3D (ULA-3D) cultures, 5000 DPCs per well were seeded in ultralow attachment 96-well plates (Corning) followed by centrifugation (200×*g*, 5 min, 4°C). SFL-3D spheroids harvested on Day 3 underwent gravity sedimentation to isolate size-defined subpopulations (40–120 μm diameter), with secondary selection via serial passaging in 96-well plates. At the designated timepoints (Days 3, 5, and 7), the spheroids were incubated with 2 μM calcein-AM/PI (Invitrogen LIVE/DEAD® Kit) for 30 min at 37°C. Fluorescent signals were captured using a Zeiss Observer microscope, where viable cells exhibited green fluorescence via esterase-mediated calcein-AM conversion, whereas membrane-compromised cells presented PI nuclear staining (red). Proliferation kinetics were analysed using CCK-8 assays. Accutase-dissociated spheroids from both systems were plated at 1000 cells/well in DMEM/F12 + 10% FBS. Following 24, 48, or 72 h of incubation, 10 μl of CCK-8 reagent was added to each well, and the absorbance at 450 nm was measured after 2 h of incubation (Tecan Infinite® M200 Pro). The growth curves were normalized to those at baseline (0 h). For the quantification of cell viability, two methods were used: automated counting using the Countess® II FL system with Trypan blue exclusion (0.4% w/v) and manual haemocytometer validation. Three technical replicates per biological sample (*n* = 4) ensured data reproducibility. Alkaline phosphatase (ALP) activity was assessed in Day 7 spheroids. Fixed samples (4% paraformaldehyde, 15 min) were incubated with the 5-Bromo-4-chloro-3-indolyl phosphate/Nitro blue tetrazolium BCIP/NBT substrate (Beyotime) for 30 min. Chromogenic signals were quantified through bright-field microscopy (Zeiss Axio Scan.Z1) and a spectrophotometric analysis (OD450) following solubilization in 1% SDS.

### Identification and evaluation of DPCs and tdDPCs

DPCs and tdDPCs were identified using established protocols [4, 10]. Specifically, the cells were incubated overnight at 4°C with LEF1 (1:100) and ALP (1:100) antibodies and then incubated for 1 h the following day with fluorescent dye-conjugated secondary antibodies. The cells were subsequently mounted with DAPI Fluoromount-G (SouthernBiotech, USA). The cells were observed using an EVOS™ FL Auto 2 microscope (Invitrogen, USA).

For the flow cytometry analysis of surface markers, DPCs and tdDPCs were incubated with PE-conjugated antibodies against CD29, CD34, CD44, CD90, and CD105 and with FITC-conjugated anti-CD45 (all at 1:20) for 30 min at 4°C. Positively stained cells were quantified using a Coulter Epics XL flow cytometer and analysed with EXPO32 ADC software.

For the differentiation assays, six-well cell culture plates were precoated with a 0.1% gelatine solution. tdDPCs were induced to undergo lipogenic differentiation at 90%–100% confluence and osteogenic differentiation at 60%–70% confluence using appropriate induction media from Cyagen Bioscience, Inc., Guangzhou, China. The cells underwent lipogenic differentiation for 2 weeks and osteogenic differentiation for 3 weeks. Induction was followed by fixation with 4% paraformaldehyde and staining with Oil Red O, alkaline phosphatase, and Alizarin Red. The staining results were examined under an Olympus IX71 light microscope (Tokyo, Japan).

Cell proliferation was assessed with a Cell Counting Kit-8 (CCK-8). DPCs at passage 5 or tdDPCs at passage 10 were dissociated with Accutase and seeded in a 96-well plate at 1000 cells per well in DMEM/F12 containing 10% FBS. On Days 0, 1, 2, 3, and 4, 10 μl of CCK-8 was added to each well and incubated at 37°C for 2 h. Cell growth curves were then obtained by measuring the absorbance at 450 nm using a Bio-Rad microplate reader.

For proliferation assays, passage 5 DPCs or passage 10 tdDPCs were seeded at a density of 10 000 cells/cm^2^ in duplicate within a 6-well plate. The number of cells was counted every day until the number of cells no longer increased. The enumeration of the cells was conducted using a Countess® automated cell counter. The average cell count obtained was plotted against the culture duration to generate a growth curve. The population doubling time (PDT) was calculated using the following formula: PDT = CT/(log(*N*/*N*_0_) × 3.33).

For ALP staining and the analysis of alkaline phosphatase activity, 1 × 10^6^ cells/cm^2^ were seeded in 12-well plates. After 24 h, the cells were fixed with 4% paraformaldehyde, washed with phosphate-buffered saline (PBS), and stained with the BCIP/NBT ALP kit for 30 min. Images were captured, and the data are displayed as the absorbance at OD450 on a microplate reader.

For the stem cell marker analysis, quantitative real-time RT–PCR was used to measure the relative mRNA expression of Nanog, Socx2, and Klf4 in DPCs and tdDPCs. RNA was extracted from cells using TRIzol and treated with RNase-free DNase according to the manufacturer’s instructions. For the analysis of mRNA expression, ~500 ng of total RNA was reverse transcribed into complementary DNA (cDNA) using the PrimeScript® RT Reagent Kit. Quantitative real-time RT–PCR was performed with the Applied Biosystems® ViiA™ 7 System using SYBR Green PCR Master Mix (TaKaRa) and 0.5 μM of each primer. Melting curves confirmed product specificity. The results are reported relative to those of GAPDH, with the primer sequences listed in Table S1 of the Supplementary Materials.

For the analysis of EV biogenesis and secretion, quantitative real-time RT–PCR was used to measure the relative mRNA expression of CD9, CD63, CD81, Alix, TSG, HRS, MVP, 1SG15, Flitillin1, KIBRA, Rab27B, Syntenin, and Syndecan1 in DPCs and tdDPCs. The specific RNA extraction, reverse transcription, and RT–PCR methods used are as described above. The results are reported relative to those of GAPDH, and the primer sequences are listed in Table S1 of the Supplementary Materials.

### EV isolation and functional validation

EVs were isolated from SFL-3D cultured tdDPCs following a 24-h incubation in medium supplemented with 10% extracellular vesicle (EV)–depleted FBS. EV isolation from the supernatant was accomplished by differential ultracentrifugation according to previous procedures [11]. The tdDPC-derived EVs (tdDPC-EVs) were subsequently quantified using the Pierce BCA Protein Assay Kit (Thermo Fisher Scientific, Waltham, MA, USA) at an absorbance of 562 nm. With the help of specific primary antibodies, we determined the expression of EV surface markers via western blotting. For the morphological analysis, the tdDPC-EVs were visualized using a transmission electron microscope (TEM; Hitachi, Japan). Further examination of their morphology and size distribution was conducted using NanoFCM, as outlined in prior studies [12]. Additionally, the tdDPC-EVs were labelled with a PKH26 red fluorescent cell linker kit (Sigma) for further analyses.

### Cell cultures

Hypertrophic scar-derived fibroblasts (HSFs) were isolated through a tissue block explant method, in which hypertrophic scar tissue was finely minced and cultured. Cultures of HSFs were maintained in DMEM (Gibco, USA) supplemented with 10% FBS (Corning, USA), 100 U/ml penicillin, and 100 μg/ml streptomycin in a humidified incubator at 37°C with 5% CO_2_. For treatment and labelling, HSFs were exposed to 20 μg/ml tdDPC-derived extracellular vesicles (tdDPC-EVs) or an equivalent volume of PBS based on the experimental group assignments. An EVOS™ FL Auto 2 microscope was used to image the PKH26-labelled tdDPC-EVs after a 24-h incubation with HSFs, followed by 30 min of fixation with 4% paraformaldehyde and staining with DAPI (D9542-1MG, Sigma–Aldrich; 1 μg/ml).

### Cell proliferation assay

HSFs were seeded into 96-well plates at a density of 5 × 10^3^ cells per well and cultured in an EV-free low-serum medium containing 2% FBS. The treatment groups included those exposed to either PBS or 20 μg/ml tdDPC-EVs. Cell proliferation was assessed using the Cell Counting Kit-8 (CCK-8; Beyotime, China), with 10 μl of the reagent added to each well at specified time points of 12, 24, 36, 48, and 60 h. Four replicates were prepared for each time point. After a 2-h incubation period at 37°C, the absorbance was measured at 450 nm to determine changes in cell proliferation.

### Scratch wound migration assay

HSFs were seeded into 6-well culture plates and allowed to reach confluence, forming monolayers. A 200-μl sterile pipette tip was used to create single straight or double crossed scratches across the cell monolayers, simulating a wound. Subsequently, floating cells were removed with PBS, and the cultures were treated with either FBS-free medium alone or FBS-free medium supplemented with EVs (20 μg/ml). The progression of wound closure was monitored and documented by photographing the scratch areas at 0 and 24 h using an inverted microscope (Zeiss, Germany). Wound areas were quantified using ImageJ (wound healing rate = [1 − (Area_T24_/Area_T0_)] × 100)%.

### Immunofluorescence staining

HSFs cultured in 6-well plates (5 × 10^4^ cells/well) were subjected to experimental treatments for 24 h prior to analysis. After treatment, the cells were fixed with 4% paraformaldehyde (Sigma–Aldrich) in PBS for 15 min at room temperature, followed by three 5-min washes with PBS. Membrane permeabilization was achieved by incubating the cells with 0.1% Triton X-100 (Thermo Fisher) in PBS for 20 min. Nonspecifically bound sites were blocked with 2% bovine serum albumin (Sigma–Aldrich) in PBS for 1 h at 25°C. Primary antibodies targeting α-smooth muscle actin (α-SMA, 1:200; Cell Signaling Technology) and collagen I (Col I, 1:200; Abcam) diluted in blocking buffer were applied overnight at 4°C. After washes with PBS-T (0.05% Tween-20), the sections were incubated with an Alexa Fluor 488–conjugated goat anti-rabbit IgG secondary antibody (1:100; Invitrogen) for 1 h at 37°C in the dark. Nuclei were counterstained with DAPI (1 μg/ml; Sigma–Aldrich) for 5 min. Fluorescence images were acquired using an Olympus FSX100 epifluorescence microscope equipped with a 20×/0.75 NA objective and standardized exposure settings. Z-Stack images (5 slices, 1 μm step size) were processed using Olympus cellSens Dimension software (v1.18) for deconvolution and maximal intensity projections.

### RNA sequencing

Total RNA was extracted from DPCs and tdDPCs or HSFs treated with tdDPC-EVs or PBS using TRIzol reagent and further purified with Dynabeads™ Oligo (dT) 25. RNA was also extracted from tdDPC-EVs. A cDNA library was constructed using the NEB kit, and transcriptome sequencing was conducted with the Illumina HiSeq Xten system. The bioinformatics analysis was performed using OmicShare by Gene Denovo Biotechnology Co. Gene expression levels were normalized to fragments per kilobase of transcript per million mapped reads (FPKM). Differentially expressed genes (DEGs) were identified using DEseq2 [13] with a false discovery rate (FDR)–adjusted *P* value <0.05 and |log2 FC| > 2.

### Lentiviral vector construction and generation of stably transfected cells

Lentiviral vectors for the overexpression and knockdown of CCNE2, along with corresponding negative control lentiviruses, were procured from VectorBuilder (Guangdong, China). HSFs were transduced with these lentiviral vectors in the presence of polybrene (GeneChem, Shanghai, China) according to the manufacturer’s protocol. After 48 h of infection, stable clonal cell lines were selected through the application of 2 μg/ml puromycin.

### miRNA interference

HSFs were cultured in 6-well plates and transfected with 100 nM synthetic RNA (miR-26a-5p mimic, inhibitor, or negative control) using Lipofectamine 2000 (Invitrogen, Carlsbad, CA, USA). A high-performance liquid chromatography method was used to purify 2-OMe-oligonucleotides that had been synthesized and labelled with carboxyfluorescein (FAM) (GenePharma, Shanghai, China) after the cells had been incubated for 24 or 48 h before analysis. Triplicate transfections and mRNA level measurements were performed.

### Quantitative reverse transcription–polymerase chain reaction

Total RNA was isolated from human scar-derived fibroblasts (HSFs) using TRIzol® reagent (Takara Bio, Japan). For mRNA quantification, 1 μg of RNA was reverse transcribed into cDNA using the PrimeScript™ RT Reagent Kit (Takara Bio). Quantitative reverse transcription–polymerase chain reaction (qRT–PCR) amplification was performed in triplicate using the UltraSYBR Mixture (CWBIO, China) on a QuantStudio 6 Flex Real-Time PCR System (Applied Biosystems), with the following thermal cycling conditions: 95°C for 10 min (initial denaturation), followed by 40 cycles of 95°C for 15 s and 60°C for 1 min. Gene expression was quantified via the 2^−ΔΔCt^ method and normalized to that of GAPDH as an endogenous control.

For miRNA profiling, 800 ng of total RNA was reverse transcribed using the Mir-X™ miRNA First-Strand Synthesis Kit (Takara Bio). Subsequent miRNA quantification was conducted with the miScript SYBR Green PCR Kit (Qiagen) and sequence-specific primers, with small nuclear RNA U6 used as the normalization reference. All reactions were executed in technical triplicates with no-template controls. The primer sequences for the target genes and miRNAs are detailed in Supplementary Table S2.

### Protein extraction and western blotting

HSFs were homogenized in ice-cold RIPA lysis buffer (Beyotime Biotechnology) supplemented with protease inhibitors. Protein concentrations were quantified using a bicinchoninic acid (BCA) assay kit (Beyotime Biotechnology). Aliquots containing equal concentrations of protein (20–50 μg) were separated by SDS–polyacrylamide gel electrophoresis and subsequently electrophoretically transferred to polyvinylidene difluoride membranes (Millipore). The membranes were blocked with 5% nonfat milk for 1 h at room temperature, followed by an overnight incubation at 4°C with primary antibodies against collagen I (Col I, 1:1000, Abcam, ab34710), α-SMA (1:1000, Cell Signaling Technology), CCNE2 (1:1000, Proteintech, 11935-1-AP), PI3K (1:1000, Proteintech, 60225-1-Ig), Akt (1:1000; Proteintech), and GAPDH (1:5000, Proteintech, 60004-1-Ig). After washing, the membranes were incubated with horseradish peroxidase (HRP)–conjugated secondary antibodies (1:5000; Proteintech) for 1 h at room temperature. The protein bands were visualized using an enhanced chemiluminescence (ECL) substrate (Millipore) and imaged with a FluorChem FC imaging system (Alpha Innotech). The quantitative densitometric analysis was performed using Image-Pro Plus software (v6.0), with GAPDH expression serving as the normalization control.

### Luciferase assay

Luciferase reporter assays were performed to verify that CCNE2 is a direct target of miR-26a-5p. Wild-type (WT) and mutant (Mut) CCNE2 3′ untranslated region (UTR) sequences were created by Genecreate (Wuhan, China). Human 293T cells were cotransfected with these plasmids and either miR-26a-5p mimics or a miR negative control (NC) using Lipofectamine 2000 (Invitrogen). After 48 h, firefly and Renilla luciferase activities were measured with a Dual Luciferase Reporter Assay Kit (Genecreate, CN).

### Animal model

A wound healing model was established in male New Zealand white rabbits (3–4 months old) under general anaesthesia induced by intravenous pentobarbital (30 mg/kg). Four full-thickness circular wounds (1 cm diameter) were surgically created on each auricle using a biopsy punch, followed by complete perichondrium excision with a scalpel [14]. After a 4-week healing period, mature crusts had formed at the wound sites. Four of these crusts were removed to photograph the underlying skin and perform a histopathological analysis of the subcrust tissue. The remaining wounds with intact crusts were subsequently randomized into four experimental groups: (1) untreated control (PBS, 100 μl); (2) tdDPC-EVs (100 μg in 100 μl of PBS); (3) agomiR-26a-5p (20 OD/ml, 100 μl); and (4) antagomiR-26a-5p (10 OD/ml, 100 μl). Starting at week 4 postwounding, subcutaneous periscar injections were administered every 2 days for 2 weeks. The longitudinal scar morphology was documented weekly. Histological evaluations, including haematoxylin and eosin (H&E) staining, Masson’s trichrome staining, Sirius red polarization microscopy, and immunohistochemical analysis of CCNE2 expression, were performed at week 4 and week 6. Digital slide scanning was conducted using an Olympus VS200 virtual microscopy system, with subsequent quantification of the scar elevation index (SEI), collagen density, collagen I/III ratio, and CCNE2 optical density using Image-Pro Plus software (v6.0).

### Statistical analysis

The data were analysed using SPSS 20. For comparisons between two groups, the *t* test was used. Comparisons of three groups over time were performed using two-way repeated measures analysis of variance (ANOVA) followed by Tukey’s *post hoc* test, whereas comparisons among three groups without time effects were performed using one-way ANOVA followed by Tukey’s *post hoc* test. The results are presented as the means ± standard deviations (SDs). A *P* value <0.05 was considered statistically significant.

## Results

### SFL-3D cultivation strategies and phenotypic characterization of tdDPCs

**Figure 1:**
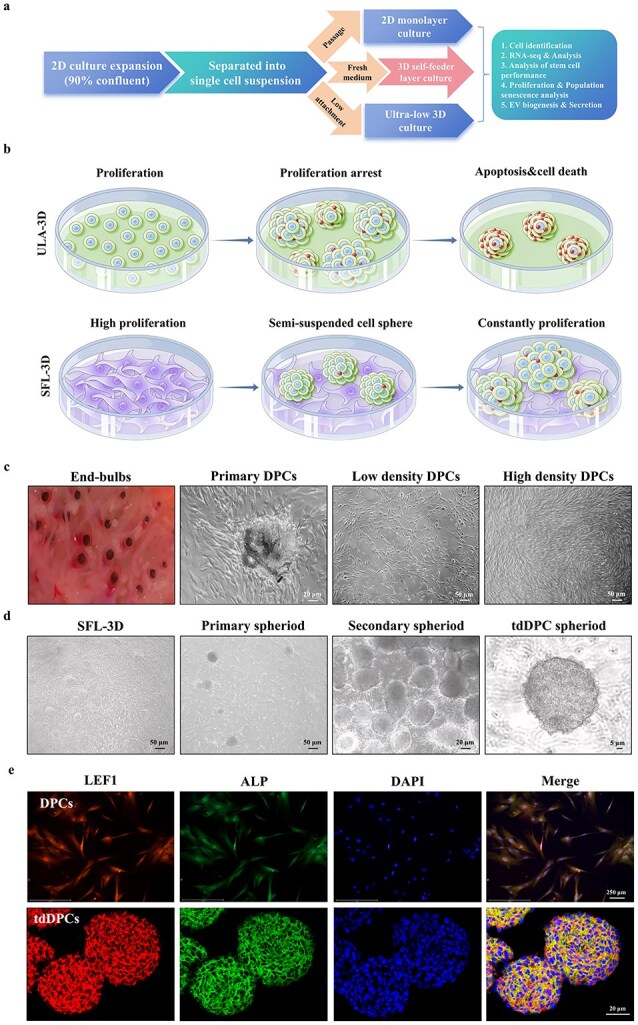
Strategies for cultivating tdDPCs using self-feeder 3D culture methods and cellular identification. (**a**) Schematic illustration of procedures from the initial 2D expansion of DPCs to the collection of 2D and SFL-3D culture media and functional analysis. (**b**) Different schematic diagrams of the ULA-3D and SFL-3D culture methods. (**c**) Culture process of primary DPCs; scale bar: 20 or 50 μm. (**d**) tdDPC SFL-3D culture process; scale bar: 5–50 μm. (**e**) Immunofluorescence staining for alkaline phosphatase (ALP) and lymphoid enhancer-binding factor 1 (LEF1), which are specific markers of DPCs or tdDPCs; scale bar: 20 or 250 μm. *DPC* dermal papilla cells, *tdDPC* three-dimensional dermal papilla cells, *ULA-3D* ultralow adhesion 3D, *SFL-3D* self-feeder layer 3D

Conventional two-dimensional (2D) culture systems for DPCs suffer from rapid replicative senescence and limited EV productivity. We developed a scaffold-free 3D culture strategy that leverages inherent proliferative heterogeneity within DPC populations to address these limitations (Figure 1a). This innovative approach enables the self-organization of highly proliferative subpopulations into semiadherent spheroids positioned above adherent DPC monolayers, designated self-feeder 3D hair follicle dermal papilla cell spheroids (SFL 3D-DPCs) or tdDPCs.

Compared with conventional scaffold-free 3D methods, the SFL-3D system demonstrated critical improvements. In contrast to ultralow attachment (ULA) dish-based cultures, where nonadherent conditions force cell aggregation into suspended spheroids (Figure 1b, upper panel), our method preserves the semiadherent architecture. ULA systems typically exhibit three limitations: (1) random spheroid fusion during prolonged culture, (2) progressive core darkening indicating centralized apoptosis (red puncta), and (3) eventual volumetric shrinkage with proliferative arrest. Conversely, SFL-3D cultures maintained structural stability through autonomous spheroid expansion rather than fusion events (Figure 1b, lower panel). The viability assessment further revealed minimal apoptotic signals in the spheroid cores, which contrasted sharply with the centralized necrosis patterns characteristic of ULA systems.

We compared cellular behaviours systematically by establishing parallel cultures of 2D-DPCs and tdDPCs according to the workflow shown in Figure 1a. The morphological analysis revealed fundamental differences: conventional 2D cultures exhibited vortex-patterned growth with contact inhibition at confluence (Figure 1c), whereas SFL-3D conditions supported hierarchical spheroid development. Primary spheroids that formed on adherent monolayers spontaneously generated secondary tdDPC structures through continuous proliferation (Figure 1d).

Molecular validation confirmed the tdDPC identity through dual immunofluorescence costaining of established dermal papilla markers. Both ALP and lymphoid enhancer-binding factor 1 (LEF1) were robustly expressed in tdDPCs (Figure 1e), with expression patterns distinct from those of their 2D counterparts. This molecular signature, combined with the enhanced proliferative capacity observed via morphological analyses, establishes tdDPCs as a functionally superior subpopulation for sustained *in vitro* expansion.

### Structural and functional characterization of SFL-3D DPC versus ULA-3D DPC spheroids

**Figure 2:**
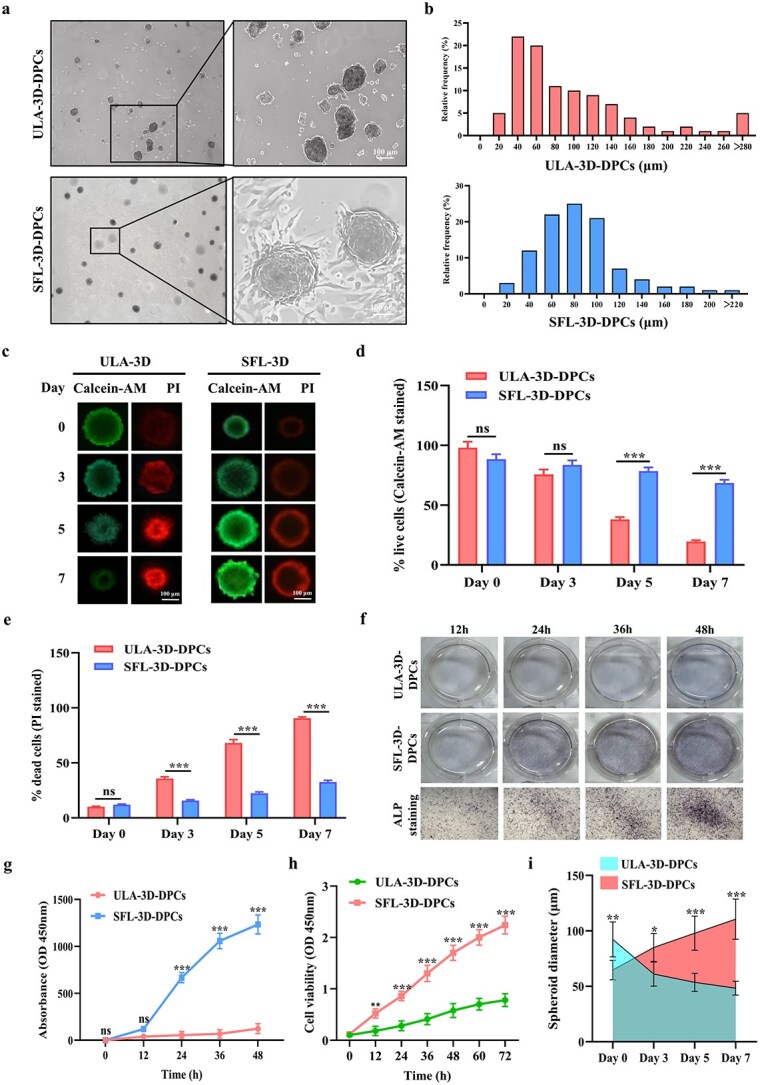
Structural and functional characterization of SFL-3D DPC *vs* ULA-3D DPC spheroids. (**a**) Comparative morphology of day 3 spheroids (phase contrast). ULA-3D—fully suspended with polydisperse sizes; SFL-3D—semisuspended with a monodispersed spherical architecture. (**b**) Stereological analysis of spheroid diameters (*n* = 5). SFL-3D—84% within 40–120 μm; ULA-3D—88% within 20–160 μm. (**c**) Representative viability profiles (days 0–7) obtained after calcein-AM and PI costaining. (**d, e**) Quantification of viable (calcein-AM+) and necrotic (PI+) cells (n=4; ^*^^*^^*^P<0.001 *vs* ULA-3D). (**f**) ALP activity as a stemness marker (Day 7). (**g**) Quantification of ALP activity (*n* = 4; ^***^P < 0.001 *vs* ULA-3D), revealing a significant increase in ALP activity in SFL-3D spheroids. (**h**) Proliferation kinetics over 72 h (*n* = 5; ^**^P <0.01 and ^***^P < 0.001). (**i**) Divergent growth trajectories: SFL-3D spheroids maintained stable expansion, while the diameter of the ULA-3D spheroids gradually decreased, and the difference was statistically significant (*n* = 5; ^*^P <0.05, ^**^P < 0.01, and ^***^P < 0.001). Scale bars: 100 μm (a, c); 500 μm (d). *ALP* Alkaline phosphatase, *DPC* dermal papilla cells, *tdDPC* three-dimensional dermal papilla cells, *ULA-3D* ultralow adhesion 3D, *SFL-3D* self-feeder layer 3D

We systematically compared the morphological and functional features of DPC spheroids generated under SFL-3D and ULA-3D culture conditions. The morphological analysis revealed distinct differences: SFL-3D spheroids exhibited semisuspended growth with a uniform size (predominantly 60–100 μm in diameter; Figure 2) and a regular spherical morphology, whereas their ULA-3D counterparts were fully suspended with irregular shapes and broader size distributions (20–160 μm; Figure 2a and b). Notably, only 1% of the SFL-3D spheroids exceeded 200 μm or fell below 20 μm, whereas 8% of the ULA-3D spheroids surpassed 280 μm (Figure 2b). Functional viability assessments further differentiated the two groups. Calcein-AM/PI staining demonstrated that the SFL-3D spheroids maintained significantly greater viability, with minimal PI-positive dead cells (Figure 2c). In contrast, ULA-3D spheroids presented a reduced calcein-AM fluorescence intensity (indicative of decreased numbers of live cells) and increased PI signals (*P *< 0.001; Figure 2d and e). An assessment of stemness by measuring ALP activity indicated that SFL-3D spheroids presented significantly higher ALP-positive cell proportions than their ULA-3D counterparts did (Figure 2f and g), which is consistent with their increased proliferative potential. Proliferation kinetics over 72 h confirmed the sustained growth advantages of SFL-3D spheroids, with cell counts consistently exceeding those of ULA-3D at all time points (Figure 2h and i). Collectively, these analyses demonstrate that compared with ULA-3D cultures, SFL-3D spheroids exhibit superior structural homogeneity, metabolic activity, stemness preservation, and proliferative vigour.

**Figure 3:**
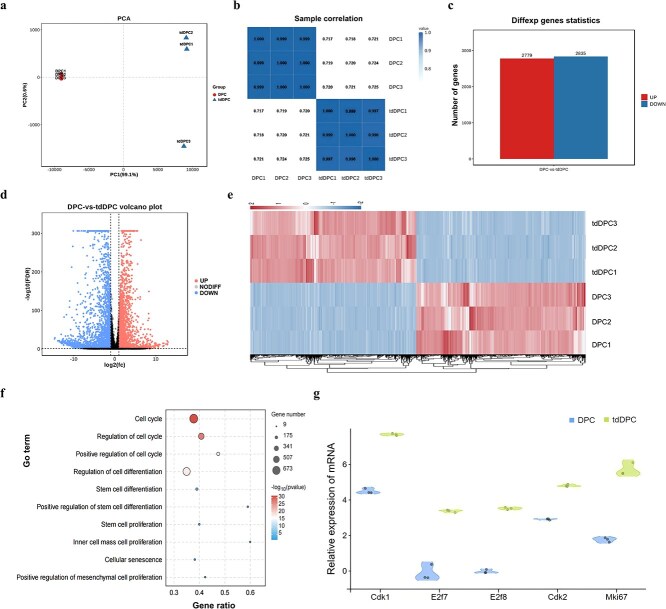
Comparative transcriptomic landscapes of DPCs and tdDPCs. (**a**) Principal component analysis (PCA) of RNA-seq data (*n* = 3 biological replicates per group). The axes represent variance contribution rates. (**b**) Pearson’s correlation matrix of transcriptomic profiles. (**c**) Statistics of the differentially expressed genes (|log2FC| ≥2, FDR <0.05). (**d**) Volcano plot of DEGs. (**e**) Heatmap of the hierarchical clustering analysis of DEGs. (**f**) Enriched GO biological process terms. The dot size reflects the gene count. (**g**) Relative mRNA expression of proliferation-associated genes. *DPC* dermal papilla cells, *tdDPC* three-dimensional dermal papilla cells, *DEGs* Differentially expressed genes, *FDR* false discovery rate, *GO* Gene Ontology

**Figure 4:**
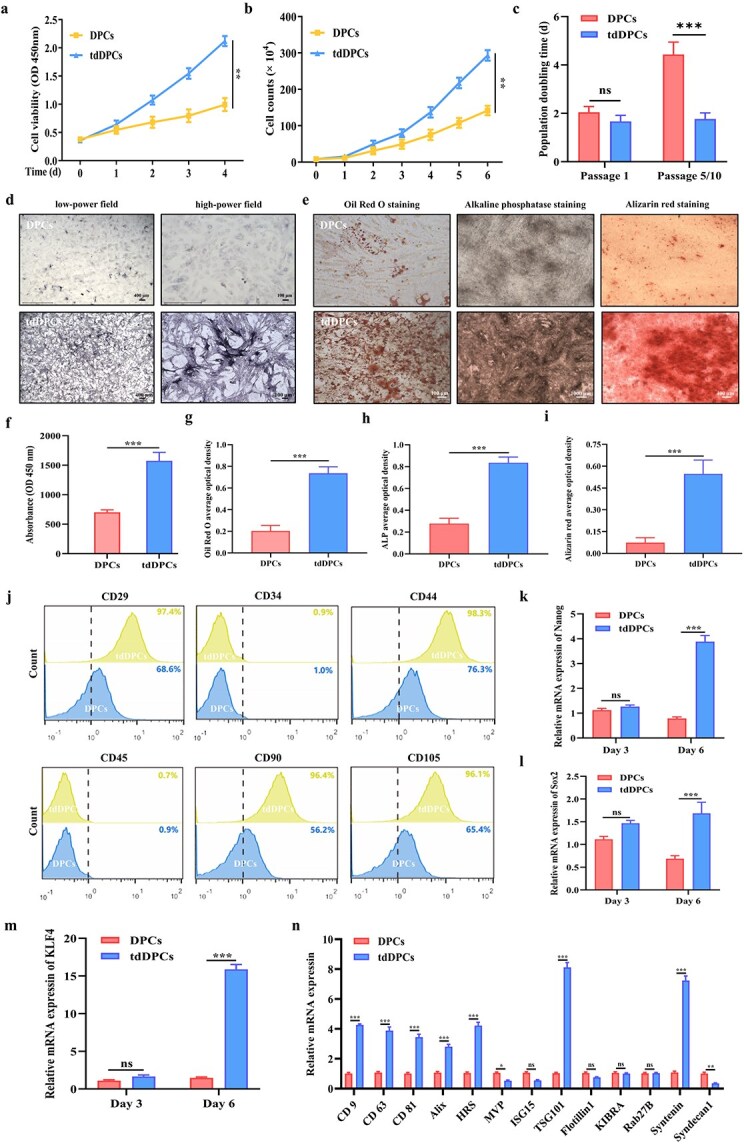
Divergent proliferative dynamics, stemness retention, and EV biogenesis characterize tdDPCs vs. DPCs. (**a**) Cell counting kit-8 (CCK-8) assay. The tdDPC cell line showed a significant increase in proliferation, *n* = 5, ^**^P < 0.01. (**b**) Plot showing the numbers of tdDPCs and DPCs, *n* = 5; ^**^P < 0.01. (**c**) Plot of the population doubling time (PDT) of tdDPC and DPC self-renewing cultures; ns, not statistically significant; *n* = 5; ^***^P < 0.001. (**d**) Passage 5 DPCs and passage 10 tdDPCs were fixed and stained with an ALP assay kit. Scale bars: 100 or 400 μm. (**e**) Comparison of the Oil Red O staining, ALP staining, and Alizarin Red staining results; scale bar: 50–500 μm. (**f**) Quantification of ALP-positive DPCs and tdDPCs at passage 5/10, *n* = 5, ^***^P < 0.001. (**g–i**) Statistical analysis of Oil Red O staining, ALP staining, and Alizarin Red staining; *n* = 5; ^***^P < 0.001. (**j**) Comparison of marker expression levels on the stem cell surface between DPCs and tdDPCs by flow cytometry. (**k–m**) qRT–PCR measurements of stem cell marker genes (Nanog, Sox2, and Klf4) in DPCs and tdDPCs. Ns, not statistically significant, *n* = 4; ^***^P < 0.001. (**n**) Expression of genes involved in EV biogenesis and secretion pathways in tdDPCs determined by qRT–PCR, *n* = 4, ^*^P < 0.5, ^**^P < 0.01, and ^***^P < 0.001. *ALP* Alkaline phosphatase, *DPC* dermal papilla cells, *tdDPC* three-dimensional dermal papilla cells

### Transcriptomic profiling and bioinformatics analysis of DPCs and tdDPCs

We performed high-throughput RNA sequencing coupled with a systematic bioinformatic analysis to delineate the transcriptome distinctions between DPCs and tdDPCs (Figure 3). Principal component analysis (PCA) revealed distinct clustering patterns between DPCs and tdDPCs, with the first two principal components explaining 78.6% of the total variance (Figure 3a). The heatmap of the intersample correlation analysis further showed high intragroup reproducibility and clear intergroup divergence (Figure 3b). The comparative analysis identified 5614 DEGs (|log2FC| ≥2, FDR-adjusted *P* ≤ 0.05), with 2779 upregulated and 2835 downregulated in tdDPCs versus DPCs (Figure 3c). The volcano plot visualization highlighted many signature genes that exceeded the threshold criteria (Figure 3d), whereas the hierarchical clustering analysis of DEGs confirmed robust separation between groups (Figure 3e). The Gene Ontology (GO) enrichment analysis performed using clusterProfiler (v4.0) revealed a significant overrepresentation (FDR < 0.05) of terms related to stem cell proliferation, differentiation, and functionality in tdDPCs (Figure 3f). Consistently, qRT–PCR validation showed 3.2- to 5.7-fold upregulation (*P* < 0.01) of the proliferation markers CDK1, CDK2, E2F7, E2F8, and MKI67 in tdDPCs (Figure 3g). These multiomics findings collectively suggest that the SFL-3D spheroid microenvironment enhances the progenitor-like properties of DPCs, increasing their proliferative and differentiation capacities.

### Divergent proliferative dynamics, stemness retention, and EV biogenesis characterize tdDPCs versus DPCs

We systematically evaluated the proliferative dynamics, stemness properties, and EV regulatory pathways in tdDPCs to comprehensively validate the transcriptome-predicted functional enhancements in these cells (Figure 4). Notably, tdDPCs maintained superior growth kinetics through 10 passages compared with early-passage (P5) DPCs, with 260% greater viability (CCK-8; Figure 4a) and a 230% greater cell yield (Figure 4b). This enhanced proliferative capacity was further evidenced by a 2.66-day decrease in the population doubling time at P10 compared with that at P5 (Figure 4c). Consistently, tdDPCs presented greater ALP activity (Figure 4d) and 54% more ALP-positive cells (Figure 4f), corroborating their enhanced stemness. Multilineage differentiation assays further revealed the superior potential of tdDPCs, with 3.6-fold greater adipogenesis (Oil Red O^+^ area), 3.0-fold greater osteogenic ALP activity (alkaline phosphatase staining), and 7.9-fold greater mineralization (Alizarin Red^+^ nodules) than DPCs (Figure 4e and g–i). The flow cytometry analysis revealed that the positive expression rate was significantly lower in passage 5 DPCs than in passage 10 tdDPCs, as illustrated in Figure 4j. Furthermore, the expression levels of the CD34 and CD45 surface markers in both DPCs and tdDPCs were <5%, indicating the absence of endothelial and haematopoietic lineage cells (Figure 4j). In addition, the qRT–PCR analysis showed delayed stemness activation: although Nanog, Sox2, and Klf4 expression did not differ on Day 3, these mRNAs were significantly upregulated in tdDPCs by Day 6 (4.3-, 2.5-, and 10.8-fold, respectively; Figure 4k–m). Given the critical role of EV-mediated signalling in DPC functionality, we quantified EV biogenesis-related genes. Compared with DPCs, tdDPCs displayed a marked upregulation of exosomal markers (CD9: 4.2-fold; CD63: 3.6-fold; CD81: 3.4-fold) and secretion regulators (Alix: 2.6-fold; TSG101: 7.9-fold; HRS: 4.0-fold; Syntenin: 6.7-fold) (Figure 4n), suggesting an increased EV-mediated regenerative potential.

### Characterization of tdDPC-EVs *vs* DPC-EVs

Having established the enhanced secretory capacity of tdDPCs, we next characterized their EV derivatives. Comprehensive characterization via transmission electron microscopy (TEM), western blotting, and nanoflow cytometry (NanoFCM) confirmed the successful isolation of tdDPC-EVs (Figure S1). TEM imaging revealed a heterogeneous population of tdDPC-EVs displaying spherical or biconcave-disk morphologies (Figure S1a). NanoFCM quantification revealed a mean particle diameter of 74.25 nm and a concentration of 5.14 × 10^10^ particles/ml (Figure S1b). Western blot analysis further validated the EV identity through positive detection of the canonical markers TSG101, CD63, and CD9 while confirming the absence of the endoplasmic reticulum contaminant calnexin (Figure S1c). Compared with DPC-EVs, tdDPC-EVs have a smaller particle size and significantly increased yield. Experimental data on the characterization and yield differences between tdDPC-EVs and DPC-EVs are provided in Figure S2 [4].

#### tdDPC-EVs inhibited the proliferation and migration of HSFs and alleviated the expression of profibrotic markers in HSFs

**Figure 5:**
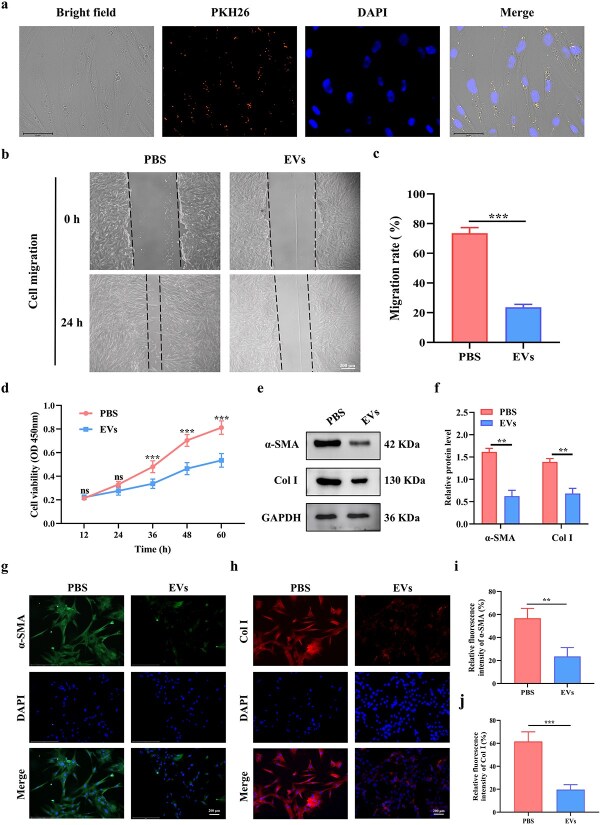
tdDPC-EVs suppress HSF activation. (**a**) Analysis of PKH26-labelled tdDPC-EV internalization by HSFs using fluorescence microscopy. (**b**) The migration of HSFs treated with tdDPC-EVs (20 μg/ml) or a similar volume of PBS was assessed through a scratch wound healing assay. (**c**) Migration rate statistics for HSFs, *n* = 5, ^***^p < 0.001. (**d**) CCK-8 assays were performed to detect proliferation after treatment; *n* = 4, n.s. not statistically significant, ^***^p < 0.001 PBS vs. EVs. (**e**, **f**) Quantification of α-SMA and Col I levels through western blot analysis, *n* = 3, ^**^p < 0.01. (**g**-**j**) Immunofluorescence staining for α-SMA and Col I in tdDPC-EV- and PBS-treated HSFs. *n* = 4, ^**^p < 0.01 and ^***^p < 0.001. Scale bar: 200 μm. HSFs hypertrophic scar fibroblasts, PBS phosphate-buffered saline, α-SMA α-smooth muscle actin, Col I collagen I, tdDPC-EVs tdDPC-derived extracellular vesicles

PKH-26-labelled tdDPC-EVs were incubated with HSFs to assess functional uptake. Fluorescence tracking revealed efficient internalization, with labelled EVs localized predominantly in the perinuclear and nuclear regions of recipient cells (Figure 5a), suggesting potential mechanisms for cargo delivery and biological activity modulation. Scratch wound healing assays demonstrated the significant inhibition of HSF migration following tdDPC-EV stimulation, with scratch closure rates reduced by 50% compared with those of the controls (Figure 5b and c). Consistent with these findings, CCK-8 proliferation assays confirmed growth suppression, with tdDPC-EV-treated HSFs exhibiting a 37% reduction in viability within 60 h (Figure 5d and Figure S3). Mechanistically, tdDPC-EVs markedly downregulated key fibrosis-associated proteins. Western blot analyses showed a 1.6-fold reduction in α-SMA levels and a 1.1-fold decrease in collagen type I (Col I) expression compared with those in PBS-treated cells (Figure 5e and f). These results were corroborated by immunofluorescence staining, which revealed that tdDPC-EV treatment diminished the α-SMA and Col I fluorescence intensities by 60% and 67%, respectively (Figure 5g–j). Notably, the coordinated attenuation of α-SMA (a hallmark of myofibroblast transdifferentiation) and Col I (a primary extracellular matrix component) expression suggests that tdDPC-EVs disrupt the fibroblast-to-myofibroblast transition, a critical driver of hypertrophic scar (HS) fibrosis. Collectively, these data establish tdDPC-EVs as potent modulators of HSF activation, highlighting their therapeutic potential to mitigate fibrotic remodelling in HS pathogenesis.

#### CCNE2 may underlie the ability of tdDPC-EVs to strongly inhibit the biological function of HSFs

**Figure 6:**
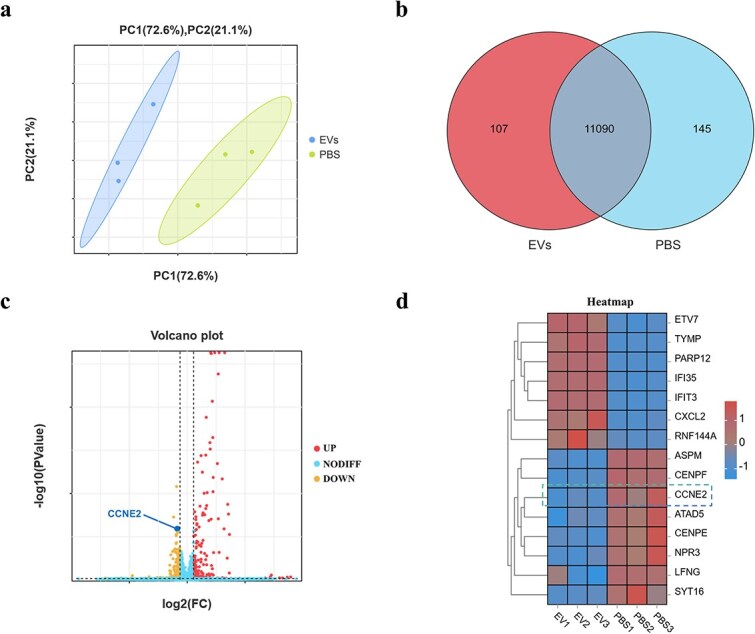
The RNA-seq and bioinformatic analysis results suggest that CCNE2 may underlie the remarkable ability of tdDPC-EVs to inhibit the biological function of HSFs. (**a**) Principal component analysis (PCA) based on gene expression information. (**b**) Venn diagram of differentially expressed genes between tdDPC-EVs and PBS. (**c**) Volcano plot of significant differences in DEG expression between tdDPC-EVs and PBS. (**d**) A grouped heatmap reflecting the comparison of differential gene expression (DiffExp) between tdDPC-EVs and PBS. *CCNE2* cyclin E2, *PBS* phosphate-buffered saline, *DEGs* differentially expressed genes, *FDR* false discovery rate, *tdDPC-EVs* tdDPC-derived extracellular vesicles

We elucidated the mechanism by which tdDPC-EVs attenuate fibrosis through the inhibition of HSF biological activity by conducting transcriptome sequencing on HSFs treated with either tdDPC-EVs or PBS. The initial PCA of global gene expression profiles revealed robust intragroup reproducibility and distinct clustering between the tdDPC-EV- and PBS-treated groups (Figure 6a), confirming treatment-specific transcriptional reprogramming. The subsequent intersection analysis via Venn diagrams identified shared and unique DEGs across the experimental conditions (Figure 6b). The volcano plot shows 119 statistically significant transcripts, comprising 82 upregulated and 37 downregulated genes (Figure 6c). Notably, CCNE2, a gene previously implicated in organ fibrosis through its role in cell cycle progression and extracellular matrix remodelling [10, 11, 15], emerged as a candidate regulator of HS. Intriguingly, CCNE2 expression was significantly lower in tdDPC-EV-treated HSFs than in PBS-treated controls (Figure 6d). This transcriptional suppression aligns with the observed inhibition of HSF proliferation and migration, suggesting a mechanistic link between CCNE2 modulation and tdDPC-EV-mediated antifibrotic effects. Collectively, these findings position CCNE2 as a pivotal molecular target through which tdDPC-EVs attenuate HSF activation and pathological fibrosis in traumatized skin. The coordinated downregulation of CCNE2 and associated profibrotic pathways provides a transcriptional framework for the therapeutic potential of tdDPC-EVs in posttraumatic scar management.

#### tdDPC-EVs suppress HSF activity via CCNE2 downregulation

**Figure 7:**
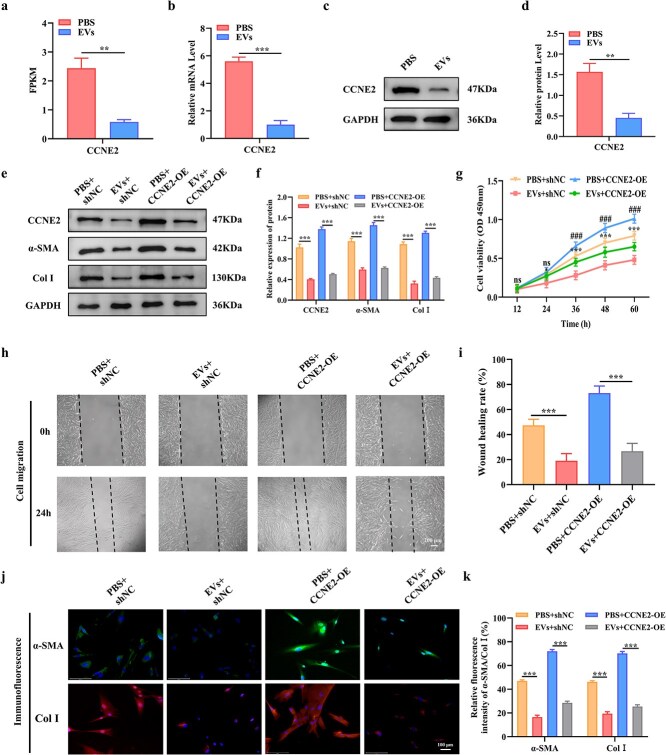
tdDPC-EVs suppress HSF fibrotic activity through CCNE2 downregulation. (**a**) RNA-seq quantification of CCNE2 expression (FPKM values). (**b**–**d**) Validation of CCNE2 suppression in tdDPC-EV-treated HSFs by qRT–PCR and immunoblotting. (**e**, **f**) Western blot analysis showing reduced CCNE2, α-SMA, and Col I levels in tdDPC-EV-treated HSFs across the experimental groups. (**g**–**i**) CCK-8 proliferation and scratch wound healing migration assays: CCNE2-OE enhances HSF activity (*P* < .05 vs. PBS + shNC), whereas tdDPC-EVs counteract this effect (###*P* < 0.001 *vs* PBS + CCNE2). Scale bars: 200 μm (*n* = 4). (**j**, **k**) Immunofluorescence staining confirming that α-SMA and Col I expression are reduced with CCNE2 suppression. Scale bars: 200 μm (*n* = 5). Ns, not statistically significant, ^*^P < 0.05, ^**^P < 0.01, and ^***^P < 0.001. *CCNE2* cyclin E2, *HSFs* hypertrophic scar fibroblasts, *PBS* phosphate-buffered saline, *α-SMA* α-smooth muscle actin, *Col I* collagen I. *CCNE2-OE* HSFs transduced with a CCNE2-overexpressing lentivirus, *tdDPC-EVs* tdDPC-derived extracellular vesicles

We performed RNA-seq followed by qRT–PCR and immunoblot validation to determine whether tdDPC-EVs mitigate fibrosis by targeting CCNE2 in HSFs, and confirmed consistent CCNE2 suppression (Figure 7a–d). Functional rescue experiments using four HSF groups—PBS + shNC, tdDPC-EVs + shNC, PBS + CCNE2-OE (overexpression), and tdDPC-EVs + CCNE2-OE—revealed that tdDPC-EVs + shNC reduced CCNE2, α-SMA, and Col I protein levels compared with those in PBS + shNC control group. Notably, compared with the PBS + CCNE2-OE group, the tdDPC-EV + CCNE2-OE group still presented reduced fibrotic protein expression (Figure 7e and f). Proliferation and migration assays showed that compared with PBS + shNC, tdDPC-EVs + shNC inhibited HSF activity, whereas PBS + CCNE2-OE enhanced HSF dynamics. Crucially, tdDPC-EVs reversed this profibrotic effect on CCNE2-OE HSFs (Figure 7g–i). The results of knockdown and rescue experiments on CCNE2 in HSFs revealed that CCNE2 knockdown decreased the expression and activity of the α-SMA and Col I proteins in HSFs, whereas CCNE2 overexpression increased the expression and activity of the α-SMA and Col I proteins in HSFs (Figure S4). Immunofluorescence staining confirmed that the reductions in the α-SMA and Col I levels paralleled CCNE2 downregulation (Figure 7j and k). These data establish CCNE2 as a central mediator of tdDPC-EV-driven HSF suppression.

**Figure 9:**
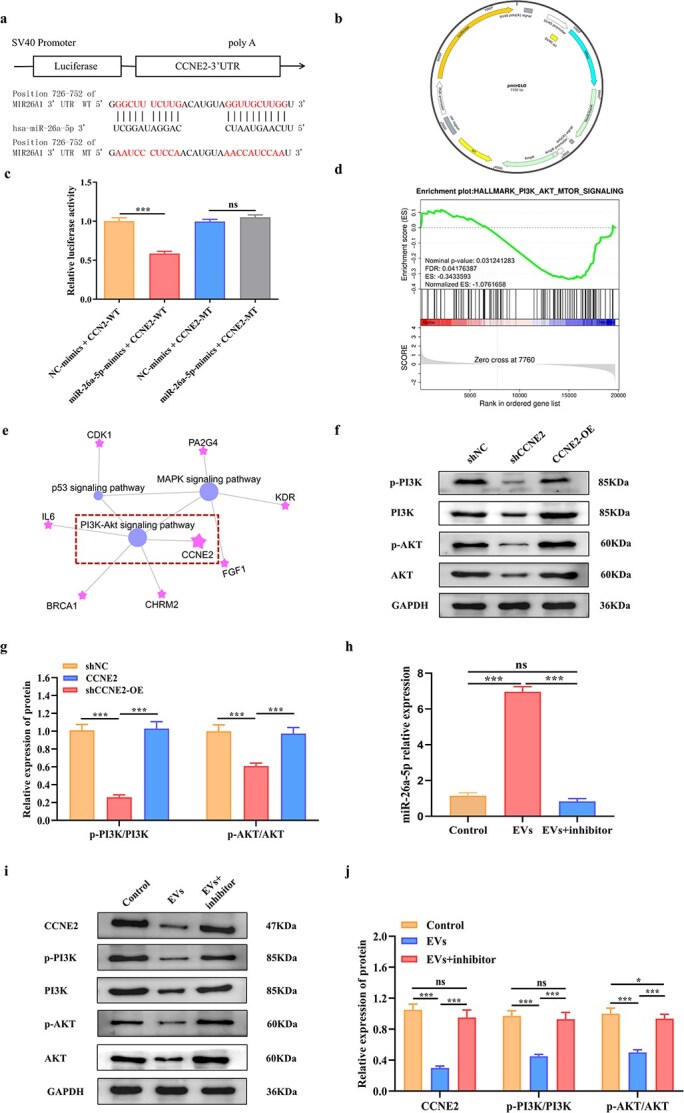
tdDPC-EVs suppress CCNE2/PI3K/AKT signalling in HSFs via miR-26a-5p delivery. (**a**) Bioinformatics prediction (TargetScan 7.0, PicTar, and miRanda) of miR-26a-5p binding sites in the CCNE2 3′UTR. (**b**, **c**) Dual-luciferase reporter assay validation: miR-26a-5p mimics significantly reduce luciferase activity in wild-type (WT) CCNE2 3′UTR-transfected 293T cells but have no effect on cells transfected with the mutant (Mut) 3′UTR constructs (*n* = 3). (**d**) GSEA showing the downregulation of the PI3K/AKT pathway in miR-26a-5p-overexpressing HSFs. (**e**) KEGG pathway analysis confirming CCNE2-PI3K/AKT functional connectivity. (**f**, **g**) Western blot quantification: CCNE2 knockdown suppresses PI3K/AKT phosphorylation, whereas CCNE2 overexpression rescues the activation of this pathway (*n* = 3). (**h**) A miR-26a-5p inhibitor reversed the tdDPC-EV-induced miRNA upregulation in HSFs (*n* = 4). (**i**, **j**) Functional rescue: cotreatment with tdDPC-EVs and the miR-26a-5p inhibitor restored CCNE2 expression and PI3K/AKT phosphorylation (*n* = 3). Ns, not significant; ^**^P < 0.01 and ^***^P < 0.001. *HSFs* hypertrophic scar fibroblasts, *GSEA* gene set enrichment analysis, *KEGG* Kyoto Encyclopedia of Genes and Genomes, *GAPDH* glyceraldehyde-3-phosphate dehydrogenase, *PI3K* phosphatidylinositol 3-kinase, *tdDPC-EVs* tdDPC-derived extracellular vesicles

#### tdDPC-EVs inhibit the biological functions of HSFs through miR-26a-5p delivery

**Figure 8:**
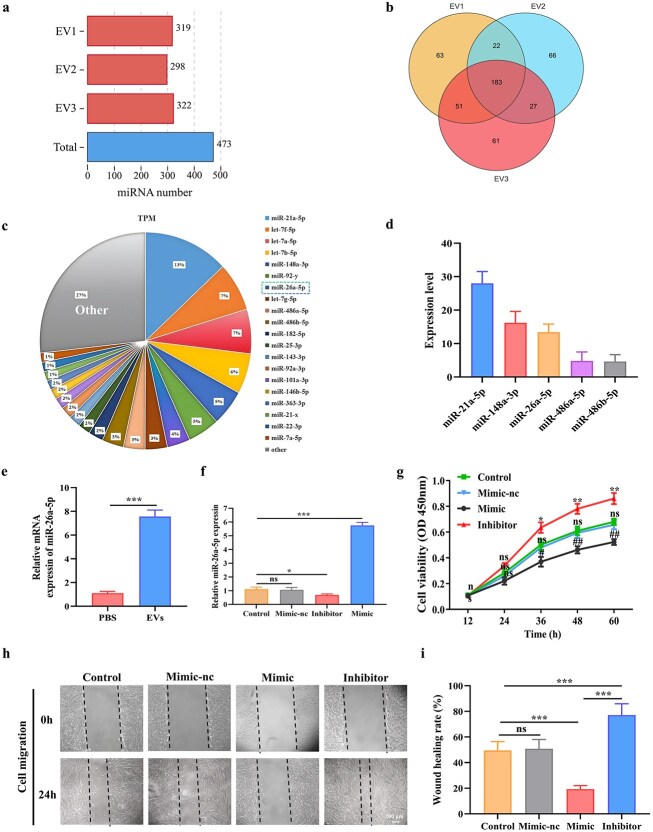
tdDPC-EVs inhibit HSF activity through targeted miR-26a-5p delivery. (**a**) miRNA profiling of tdDPC-EVs across experimental groups. (**b**) Venn diagram identifying 183 conserved miRNAs shared among all groups. (**c**) Relative abundance of the top 20 miRNAs (>70% total content), including miR-26a-5p and let-7 family members. (**d**) qRT–PCR validation of selected miRNAs (miR-21a-5p, miR-148a-3p, miR-26a-5p, and miR-486a-5p/b-5p) in tdDPC-EVs. (**e**) miR-26a-5p upregulation in HSFs following tdDPC-EV treatment compared with that in PBS controls. (**f**) Efficacy of miR-26a-5p mimic/inhibitor transfection: Mimics increase expression, whereas inhibitors decrease endogenous levels. (**g**–**i**) Functional validation: miR-26a-5p mimics recapitulate the tdDPC-EV-mediated suppression of HSF proliferation (CCK-8 assay, g) and migration (scratch wound healing assay, **h**, **i**), whereas inhibitors reverse these effects, *n* = 4, scale bar: 200 μm; ^*^P < 0.05, ^**^P < 0.01, and ^***^P < 0.001. *HSFs* hypertrophic scar fibroblasts, *tdDPC-EVs* tdDPC-derived extracellular vesicles

As critical functional components of EVs, miRNAs play pivotal roles in intercellular communication. We first performed a microarray analysis to profile the miRNA cargo in tdDPC-EVs and identify the miRNAs that mediate the effects of tdDPC-EVs on HSFs. The Venn diagram shows 183 conserved miRNAs across all the tdDPC-EV groups (Figure 8a and b), with the top 20 most abundant miRNAs (e.g. miR-21a-5p, let-7 family members, and miR-26a-5p) accounting for >70% of the total miRNA content (Figure 8c). Bioinformatic target prediction (OmicsMart platform) identified CCNE2 as a top candidate target of miR-26a-5p, linking this miRNA to our previously observed antifibrotic effects. qRT–PCR confirmed the enrichment of miR-26a-5p in tdDPC-EVs (Figure 8d) and its significant upregulation in HSFs following tdDPC-EV treatment (Figure 8e). We transfected HSFs with miR-26a-5p mimics or inhibitors to establish functional causality. While mimic-negative controls (mimic-nc) had no effect, mimics robustly elevated miR-26a-5p levels and inhibitors suppressed endogenous expression (Figure 8f). Subsequent functional assays demonstrated that the miR-26a-5p mimics reversed the tdDPC-EV-mediated suppression of HSF proliferation (CCK-8 assay, Figure 8g) and migration (scratch wound healing assay, Figure 8h and i). Conversely, miR-26a-5p inhibition exacerbated HSF activity, confirming bidirectional regulation. Notably, the anti-proliferative/migratory effects of tdDPC-EVs correlated directly with the miR-26a-5p transfer efficiency (Figure 8e vs. 8g–i). These findings collectively indicate that tdDPC-EVs attenuate HSF pathological behaviours through the targeted delivery of miR-26a-5p, which mechanistically suppresses profibrotic signalling via CCNE2 downregulation.

#### tdDPC-EVs downregulate CCNE2 and the PI3K/AKT pathway in HSFs via miR-26a-5p delivery

We first performed comprehensive bioinformatics analyses using TargetScan 7.0, PicTar, and miRanda to elucidate whether miR-26a-5p suppresses HSF activity by targeting CCNE2. These algorithms collectively predicted a conserved binding site for miR-26a-5p in the 3′UTR of CCNE2 (Figure 9a). Subsequent validation via dual-luciferase reporter assays in 293T cells showed that miR-26a-5p mimics significantly reduced luciferase activity in cells transfected with the wild-type CCNE2 3′UTR. Notably, this suppression was abolished when mutant CCNE2 3′UTR constructs were used (Figure 9b and c), confirming the direct interaction between miR-26a-5p and the CCNE2 3′UTR. Gene set enrichment analysis (GSEA) of miR-26a-5p-overexpressing HSFs revealed the significant downregulation of PI3K/AKT signalling (Figure 9d), whereas the KEGG pathway analysis further established functional connectivity between CCNE2 and PI3K/AKT signalling (Figure 9e). We modulated CCNE2 expression in HSFs and assessed PI3K/AKT pathway activation to validate these findings. Strikingly, CCNE2 knockdown suppressed PI3K/AKT phosphorylation (^*^P < .05 vs. the control), whereas CCNE2 overexpression rescued this activation (Figure 9f and g), establishing CCNE2 as a key regulator of PI3K/AKT signalling. We treated HSFs with tdDPC-EVs combined with miR-26a-5p inhibitors to determine whether tdDPC-EVs mediate these effects through miR-26a-5p delivery. While tdDPC-EVs alone significantly reduced CCNE2 expression, cotreatment with inhibitors partially restored CCNE2 levels (Figure 9h–j). Consistently, tdDPC-EVs suppressed PI3K/AKT phosphorylation, and this inhibition was reversed by the miR-26a-5p inhibitor (Figure 9i and j). Collectively, these data indicate a mechanistic cascade in which tdDPC-EVs deliver miR-26a-5p to HSFs, directly targeting the CCNE2 3′UTR to suppress its expression, thereby inhibiting PI3K/AKT pathway activation, and ultimately attenuating HSF pathological activity.

#### miR-26a-5p reduces HS and improves wound healing quality by targeting CCNE2

**Figure 10:**
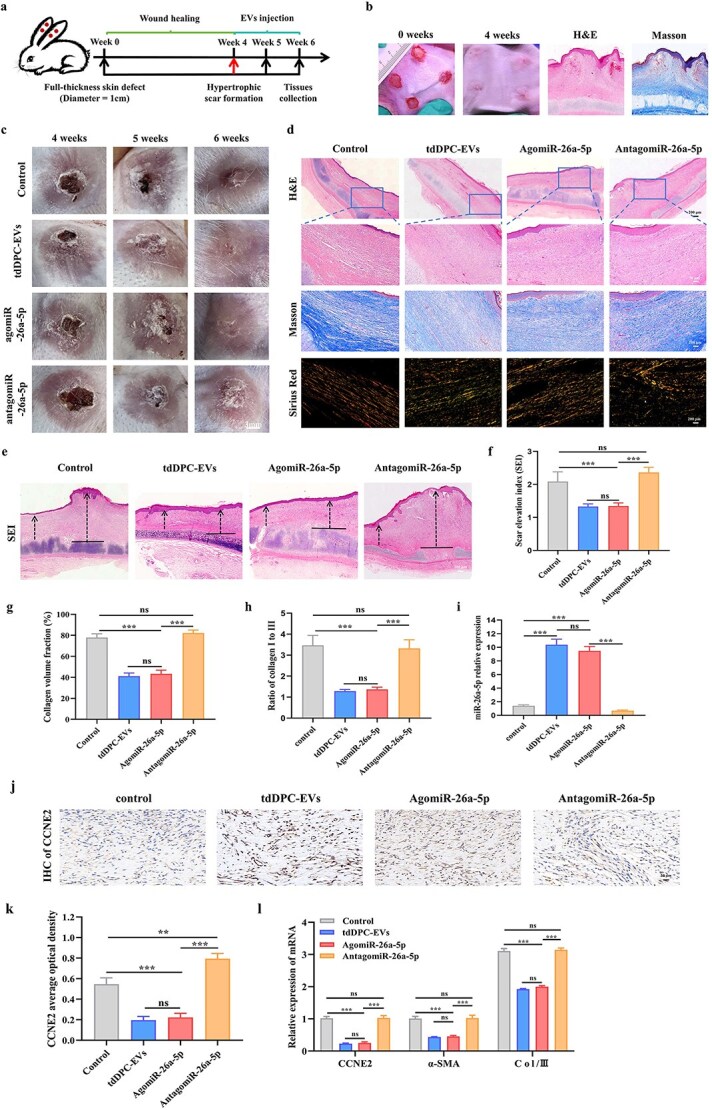
miR-26a-5p mimics tdDPC-EVs in suppressing hypertrophic scarring through CCNE2 regulation. (**a**) Flow chart of the treatment time and progress of wound healing from week 0 (immediately after injury) to week 6. (**b**) H&E staining of 4-week-old HS lesions showing epidermal hyperplasia, inflammatory clusters, and dermal thickening. Scale bar: 200 μm. (**c**) Gross morphology of the wound healing process from week 4 to week 6. (**d**) H&E, Masson’s trichrome, and Sirius red staining of HS maturation at week 6. Scale bars: 50–200 μm. (**e**–**f**) Quantification of the scar elevation index (SEI) and dermal thickness (*n* = 8). (**g**–**h**) Collagen reorganization was assessed by Masson’s trichrome and Sirius red (birefringence) staining. Collagen I/III ratio quantified under polarized light. (**i**) miR-26a-5p expression was determined by qRT–PCR (normalized to U6). (**j**) Representative images of IHC staining showing CCNE2 expression. Scale bar: 50 μm. (**k**) Analysis of CCNE2 expression in skin at 6 weeks in various treatment groups (*n* = 8). (**l**) mRNA expression of CCNE2, α-SMA, and Col I/III in each treatment group (*n* = 4). Ns, not significant; ^***^P < 0.001. *H&E* haematoxylin and eosin, *GAPDH* glyceraldehyde-3-phosphate dehydrogenase, *α-SMA* α-smooth muscle actin, *Col I/III* collagen I/III, *tdDPC-EVs* tdDPC-derived extracellular vesicles

We established a rabbit ear scar model and administered four treatments at 4 weeks postinjury to assess the therapeutic potential of miR-26a-5p for HS: PBS (control), tdDPC-EVs, agomiR-26a-5p, and antagomiR-26a-5p. Gross morphological changes were documented weekly from weeks 4 to 6 (Figure 10a). The successful induction of HS at week 4 was confirmed by a histopathological analysis. The histological evaluation at the 4-week maturation phase, corresponding to the peak developmental stage of hypertrophic scarring in the rabbit ear model, revealed fully formed subeschar hypertrophic scar tissue upon eschar removal. This tissue exhibited characteristic features, including epidermal and dermal hyperplasia, neovascularization, and inflammatory cell infiltration, as shown in Figure 10b and c. At week 6 posttreatment, the tdDPC-EV and agomiR-26a-5p groups presented significant scar resolution, with a pliable, noncontracted tissue morphology (SEI: 1.3 ± 0.1 vs. 2.3 ± 0.2 in the control group). In contrast, the PBS- and antagomiR-26a-5p-treated groups retained rigid, hyperkeratotic scars (Figure 10d–f). Histologically, the therapeutic interventions reduced the dermal thickness by 44% (Figure 10f). The collagen analysis revealed decreased total collagen deposition and normalized collagen I/III ratios following tdDPC-EV or agomiR-26a-5p treatment (Figure 10g and h). Mechanistically, qRT–PCR indicated a 5.1-fold miR-26a-5p upregulation with concomitant 65% CCNE2 downregulation in the therapeutic groups (Figure 10i–k). Crucially, the expression of CCNE2, α-SMA, or Col I/III was notably reduced in response to agomiR-26a-5p, and a significant positive correlation was observed between α-SMA and Col I/III levels and CCNE2, similar to our *in vitro* observations (Figure 10i). These data establish miR-26a-5p as a functional mimic of tdDPC-EVs that attenuates HS progression and improves the quality of wound healing *in vivo* by targeting CCNE2.

## Discussion

HS following traumatic injury is a prevalent complication of aberrant wound healing, with posttraumatic HS representing a severe fibroproliferative disorder characterized by a disrupted skin architecture and functional impairment [16]. Despite advances in therapeutic strategies, the effective prevention and management of posttraumatic HS remain clinically challenging. These findings underscore the urgent need for innovative interventions to mitigate fibrotic progression and optimize tissue regeneration. In this study, we developed SFL-3D to engineer tdDPCs with enhanced proliferative potential, increased stemness properties, and increased EV production. Subsequently, we utilized EVs derived from tdDPCs to treat HSFs or HS tissues. Comprehensive functional analyses *in vivo* and *in vitro* showed that tdDPC-EVs reduce HS after trauma through the miR-26a-5p/CCNE2/PI3K/AKT signalling pathway, thereby improving the quality of wound healing.

Emerging evidence has demonstrated that hair follicle–derived stem cells ameliorate HS through the multifaceted modulation of the wound microenvironment, attenuating inflammatory cascades, orchestrating paracrine signalling, and steering fibroblast lineage commitment towards regenerative phenotypes [17]. Of particular therapeutic relevance, DPCs exhibit direct reparative plasticity by differentiating into functional dermal fibroblasts that actively participate in wound matrix remodelling, thereby mitigating posthealing fibrosis [18]. This regenerative capacity has been successfully harnessed in bioengineered DPC-based skin substitutes, which recapitulate the native tissue architecture through epidermal basement membrane regeneration while concurrently suppressing pathological wound contraction, achieving biomechanical properties (elasticity and tensile strength) approximating uninjured skin [19]. Notably, these constructs further exhibit scar-suppressive synergy through two mechanisms: inducing mature vasculature networks to enhance nutrient perfusion and resolving inflammatory niches through cytokine rebalancing, collectively inhibiting fibrotic progression and wound shrinkage [20].

While conventional 2D culture systems induce progressive functional deterioration in primary DPCs, characterized by a hypertrophic morphology, premature senescence, and proliferative arrest, these limitations critically impair their hair-inductive capacity [21, 22]. The 3D spheroid culture has emerged as a regenerative strategy to restore DPC functionality and to circumvent this biological constraint. Compelling evidence has shown that 3D-cultured DPC spheroids not only reacquire a superior hair follicle–inductive potential compared with their monolayer counterparts but also exert paracrine-mediated therapeutic effects beyond localized administration sites, suggesting the systemic modulation of dermal regenerative niches [23]. Contemporary 3D culture systems for mesenchymal stem cells (MSCs) predominantly employ scaffold-free methodologies, particularly ULA-3D platforms, which force cellular aggregation into spheroids by inhibiting substrate attachment [24]. However, these conventional nonscaffold approaches face critical limitations: prolonged culture induces central necrosis due to oxygen/nutrient diffusion barriers, accelerates proliferative decline (evidenced by prolonged population doubling times), and fails to maintain cellular heterogeneity—challenges exacerbated in DPCs that inherently exhibit senescence susceptibility and viability fluctuations [25].

We developed a scaffold-free self-feeder layer 3D (SFL-3D) system harnessing the intrinsic proliferation heterogeneity of DPCs to overcome these constraints. This innovation enables autonomous stratification: highly proliferative subpopulations form semiadherent spheres atop wall-adherent counterparts, eliminating exogenous scaffolds while maintaining structural stability through dual mechanisms: (1) spontaneous aggregation of mitotically active DPCs under optimized culture conditions and (2) matrix scaffolding by adherent DPCs that secrete prosurvival factors and provide anchor points for sphere attachment. The SLF-3D system generates spheroids with a sustained proliferative capacity through a unique microenvironmental design that eliminates the artificial physicochemical interventions required by conventional 3D methods. Unlike surface material–dependent approaches, where the lack of substrate adhesion may alter cellular homeostasis, our semisuspended culture mechanism prioritizes medium optimization—specifically, the synergistic action of DPC-secreted growth factors and exosomes within a biomimetic matrix. This configuration enables the autonomous maintenance of progenitor cell viability while preserving native cell–cell signalling architectures. In this study, for the first time, we systematically compared the differences in structural homogeneity, metabolic activity, stemness preservation, proliferative vigour, directed induction of differentiation, and vesicle secretion ability between DPCs cultured with SFL-3D and ULA-3D and DPCs cultured in 2D. Crucially, SFL-3D-cultured tdDPCs displayed a sustained expansion capacity (>10 passages) without expressing senescence markers, which contrasted sharply with 2D-cultured DPCs, which exhibited accelerated ageing by passage 5. Transcriptomic profiling revealed increased expression of stemness markers and upregulated EV biogenesis-related gene expression in tdDPCs, corroborating the findings from adipose-derived MSC studies using analogous platforms [26, 27]. This increase in EV production directly addresses the low vesicle yield of conventional DPC cultures, establishing a robust foundation for harvesting therapeutically potent EVs.

While tdDPCs exhibit therapeutic potential, the inherent limitations of cell-based therapies, including immunogenicity concerns, tumorigenic risks, and challenges in clinical-scale delivery, motivated our focus on their EVs. As natural nanoscale mediators of intercellular communication, EVs retain parental cell bioactivity while overcoming translational barriers through reduced immunogenicity, an enhanced biodistribution, and improved storage stability [28, 29]. Our engineered tdDPCs exhibit increased EV production with optimized regenerative cargo profiles, overcoming the critical limitations of conventional MSC-EV therapies related to yield and the functional molecular composition [4, 5]. This strategic pivot to tdDPC-EVs harnesses essential therapeutic mechanisms while eliminating the risks associated with whole-cell administration.

HS pathogenesis is driven by the fibroblast-to-myofibroblast transdifferentiation, which is characterized by excessive collagen deposition and α-SMA-mediated contractile force generation [29–31]. While myofibroblasts facilitate physiological wound closure through transient collagen synthesis [32], their persistent activation induces pathological matrix remodelling, manifesting as elevated Col I production and tissue contracture, which are hallmark features of HS. HSFs, which exhibit myofibroblast-dominant phenotypes (fibronectin ED-A+, α-SMA+, and Col I hypersecretion), were employed as a pathological model [33, 34]. Our investigations revealed that tdDPC-EVs effectively attenuated HS progression through two mechanisms: (1) inhibiting the HSF proliferative capacity and migratory potential and (2) downregulating the expression of fibrotic markers. Confocal microscopy confirmed rapid tdDPC-EV internalization by HSFs within 24 h, which was correlated with functional suppression. Notably, while these findings establish tdDPC-EVs as potent antifibrotic agents, the precise molecular mediators governing their therapeutic effects remain to be fully elucidated.

CCNE2, a cyclin-dependent kinase regulator, has emerged as a critical mediator of fibrotic matrix deposition across multiple organ systems [11, 35, 36]. RNA sequencing revealed that tdDPC-EV treatment markedly suppressed CCNE2 expression in HSFs, indicating its therapeutic relevance. qRT–PCR and immunoblot analyses validated CCNE2 downregulation in tdDPC-EV-treated HSFs. We engineered CCNE2-overexpressing HSFs via lentiviral transduction to establish causality. Intriguingly, compared with scrambled shRNA controls, tdDPC-EVs retained their antifibrotic efficacy in CCNE2-overexpressing cells, significantly reducing the protein levels of both CCNE2 and its downstream effectors, Col I and α-SMA. Rescue experiments confirmed that CCNE2 suppression mediated the tdDPC-EV-induced inhibition of HSF proliferation and migration. These findings mechanistically position CCNE2 as a nodal regulator through which tdDPC-EVs mitigate pathological matrix remodelling. 

Emerging paradigms position EV-mediated miRNA transfer as a critical regulator of fibrotic signalling cascades [26]. Our multiomics integration (RNA-seq/miRNA-seq) identified miR-26a-5p as the predominant tdDPC-EV cargo. This mechanistic divergence prompted a systematic evaluation of alternative miRNA regulators. Bioinformatic triangulation (TargetScan and miRDB) revealed that miR-26a-5p is the sole tdDPC-EV-enriched miRNA (top decile abundance) with a conserved binding capacity to the CCNE2 3′UTR. Luciferase reporter assays confirmed the sequence-specific interaction between miR-26a-5p and CCNE2. Strikingly, miR-26a-5p inhibition abrogated the tdDPC-EV-mediated suppression of HSF fibrogenic activity, whereas CCNE2 overexpression reversed the antifibrotic effects. Intriguingly, the residual suppression of fibrotic markers (α-SMA and collagen I) in CCNE2-overexpressing HSFs suggests that tdDPC-EVs engage auxiliary pathways to amplify therapeutic outcomes—a phenomenon that is consistent with the emerging concept of EV-mediated ‘network pharmacology’ in fibrosis resolution. Notably, this regulatory axis aligns with conserved miR-26a-5p-mediated fibrosis modulation in pulmonary/cardiac systems [37–39] but constitutes its first mechanistic demonstration in cutaneous scarring. Future studies should deconvolute the full EV cargo repertoire (e.g. protein, lncRNA, and lipids) and dissect their interplay with host signalling hubs (e.g. TGF-β/SMAD and Wnt/β-catenin) to map the complete regulatory topology. Our findings establish that EV-packaged miR-26a-5p is the dominant effector of tdDPC-EV bioactivity through direct CCNE2 epistasis while underscoring the need to characterize complementary mechanisms that confer resistance to target pathway perturbations.

The PI3K/AKT signalling axis, a master regulator of fibrogenic activation, drives pathological matrix remodelling in HS through sustained myofibroblast survival and differentiation [40–44]. Emerging pharmacological evidence positions CCNE2 as an upstream modulator of PI3K/AKT phosphorylation dynamics, although its mechanistic interplay with EV-derived miRNAs remains unexplored [45, 46]. Integrative bioinformatics (KEGG pathway enrichment analysis and miRanda target prediction) revealed a strong inverse correlation between miR-26a-5p enrichment in tdDPC-EVs and CCNE2/PI3K/AKT pathway activation. Functional validation showed that CCNE2 knockdown in HSFs suppressed PI3K and AKT phosphorylation, whereas CCNE2 overexpression reversed this inhibition. Crucially, tdDPC-EV treatment upregulated miR-26a-5p expression concomitant with CCNE2 downregulation and PI3K/AKT dephosphorylation. This antifibrotic effect was abolished by miR-26a-5p inhibition, establishing miR-26a-5p as the dominant EV cargo mediating PI3K/AKT inactivation via CCNE2 targeting. These findings delineate a novel miR-26a-5p/CCNE2/PI3K/AKT regulatory axis in HS pathogenesis, representing the first evidence of EV-mediated epigenetic regulation of this profibrotic cascade. This pathway differs from previously reported miRNA-mediated antifibrotic mechanisms, such as miR-29 targeting of TGF-β/SMAD or miR-145 modulating ECM remodelling, by uniquely converging on CCNE2-mediated cell cycle regulation and mechanotransduction pathways.

We employed a well-established rabbit ear wound model in which mature HSs typically develop by week 4 after trauma to investigate the role of miR-26a-5p *in vivo* [14]. Systemic interventions were initiated at this critical phase, corresponding to the transition from the proliferative stage to the remodelling stage, to evaluate antifibrotic efficacy in histologically stabilized scars. Experimental validation revealed that the local delivery of agomiR-26a-5p effectively ameliorated hypertrophic scar formation, as evidenced by attenuated scar elevation and collagen matrix disorganization. This phenotypic improvement correlated with the marked suppression of α-SMA expression—a hallmark of myofibroblast activation in fibrotic pathologies [47, 48]—mirroring the antifibrotic effects previously observed with tdDPC-EV treatment. Mechanistically, consistent with our *in vitro* findings, miR-26a-5p administration suppressed both CCNE2 and collagen I/III production, whereas pharmacological inhibition via antagomiR-26a-5p reversed these therapeutic benefits and exacerbated pathological progression. Crucially, the coordinated regulation of this molecular cascade confirms that tdDPC-EVs mitigate fibrosis through the targeted modulation of the miR-26a-5p/CCNE2 axis, subsequently disrupting PI3K/AKT-driven fibrogenic signalling. Collectively, these data establish a causal link between EV-derived miR-26a-5p and the multitiered inhibition of mechanofibrotic circuits *in vivo*.

At present, the treatment of HSs with corticosteroids and silicone gel primarily alleviates symptoms but fails to address the root cause of fibroblast dysfunction. In contrast, miR-26a-5p-enriched tdDPC-EVs achieve dual inhibition. This coordinated action aligns with emerging strategies targeting fibrotic signalling hubs, such as PI3K/AKT inhibitors, in pulmonary fibrosis but with increased specificity due to EV-mediated miRNA delivery. Although clinical trials of treatments targeting CCNE2 in HS have not yet been conducted, the SFL-3D platform has increased the production of DPC-derived EVs and stably carried miR-26a-5p, solving a key bottleneck in the manufacturing of miRNA therapies and laying the foundation for future clinical trials. Further studies should explore combinatorial strategies, such as coupling miR-26a-5p delivery with PI3K/AKT inhibitors or mechanomodulatory agents, to amplify the antifibrotic effects. Additionally, validating the safety and biodistribution of tdDPC-EVs in large animal models will bridge the gap in clinical translation. Moreover, other limitations of this study should be acknowledged. Although miRNAs are critical mediators of tdDPC-EV bioactivity, other components may synergistically contribute to the antifibrotic effects. Future studies also require a systematic analysis of the proteomic and lncRNA profiles of tdDPC-EVs to identify complementary therapeutic agents and the mechanisms by which they exert their antifibrotic effects.

## Conclusions

In summary, our study establishes that the SFL-3D culture system uniquely enhances the DPC proliferation potency, stem cell functional integrity, and EV productivity compared to conventional monolayer conditions. Crucially, tdDPC-derived EVs execute multitarget antifibrotic effects through the miR-26a-5p-mediated silencing of CCNE2, effectively suppressing PI3K/AKT signalling to concurrently mitigate collagen hyperproduction, the fibroblast-to-myofibroblast transdifferentiation, and hypertrophic scar progression. These findings validate the SFL-3D platform as an innovative strategy for the scalable production of therapeutic EVs while delineating a definitive miR-26a-5p/CCNE2 regulatory axis that bridges EV cargo delivery with the epigenetic control of fibrotic cascades. Our work provides a blueprint for exploiting 3D-cultured stromal cell derivatives as next-generation acellular therapeutics for pathological scarring.

## Abbreviations

ALP: alkaline phosphatase; agomiR-26a-5p: miR-26a-5p agomir; antagomiR-26a-5p: miR-26a-5p antagomir; α-SMA: α-smooth muscle actin; BCA: bicinchoninic acid; Col I: collagen I; CCNE2: cyclin E2; DEGs: differentially expressed genes; DAPI: 4′6-diamidino-2-phenylindole; DMEM: Dulbecco’s modified Eagle’s medium; DPCs: dermal papilla cells; ECL: enhanced chemiluminescence; EVs: extracellular vesicles; FBS: foetal bovine serum; FITC: fluorescein isothiocyanate; FPKM: fragments per kilobase of transcript per million mapped reads; FDR: false discovery rate; FC: fold change; GSEA: gene set enrichment analysis; H&E: haematoxylin and eosin; HRP: horseradish peroxidase; HS: hypertrophic scar; HSFs: hypertrophic scar fibroblasts; KEGG: Kyoto Encyclopedia of Genes and Genomes; IGF-1: insulin-like growth factor 1; IHC: immunohistochemical; LEF1: lymphoid enhancer-binding factor 1; MSCs: mesenchymal stem cells; miRNA: microRNA; miR: microRNA; OD: optical density; PI3K: phosphatidylinositol 3-kinase; PDGF: platelet-derived growth factor; PE: phycoerythrin; qRT–PCR: quantitative reverse transcription–polymerase chain reaction; RNA-seq: RNA sequencing; shRNA: short hairpin RNA; SFL-3D: self-feeder layer 3D; TEM: transmission electron microscopy; TSG101: tumour susceptibility gene 101; tdDPCs: three-dimensional dermal papilla cells; tdDPC-EVs: tdDPC-derived extracellular vesicles; UTR: untranslated region; ULA-3D: ultralow adhesion 3D; GO: Gene Ontology.

## Supplementary Material

Figure_S1_tkaf048

Figure_S2_tkaf048

Figure_S3_tkaf048

Figure_S4_tkaf048

Table_S1_tkaf048

Table_S2_tkaf048

supplementary1_tkaf048

## Data Availability

The data used to support the findings of this study are available from the corresponding author upon request.
