# Supplementary material for: Self-organizing three-dimensional dermal papilla cell spheroids yield therapeutic extracellular vesicles that target hypertrophic scar regression via the miR-26a-5p/CCNE2 axis
Source: Burns Trauma. 2025 Jul 22;14:tkaf048. doi: 10.1093/burnst/tkaf048 (PMC13345373; doi:10.1093/burnst/tkaf048)
Supplement: Figure_S1_tkaf048 [file figure_s1_tkaf048.docx]

**Figure S1**


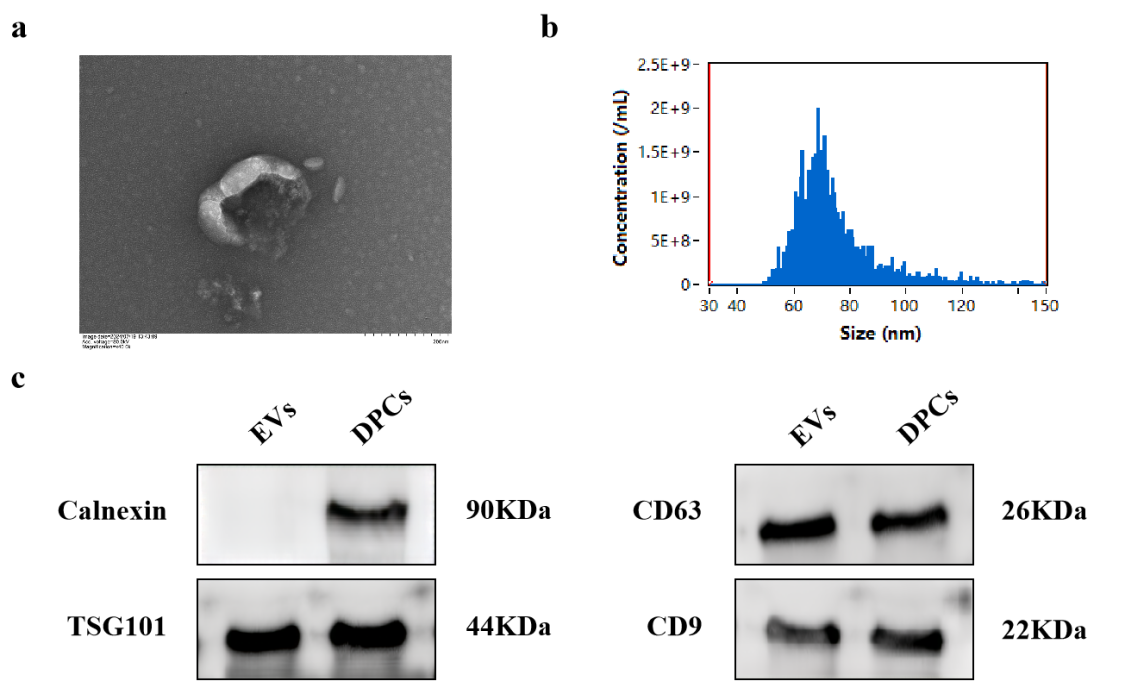


**Figure S1**. Characterization and target cell internalization of 3D dermal papilla cells (tdDPC-EVs). (a) Ultrastructure of tdDPC-EVs visualized via transmission electron microscopy (TEM); scale bar: 200 nm. (b) Particle size distribution of tdDPC-EVs determined using NanoFCM. (c) Analysis of the EV markers TSG101, calnexin, CD63, and CD9 through Western blotting. DPC lysates served as a control. Scale bar: 125 μm.
