# Supplementary material for: Self-organizing three-dimensional dermal papilla cell spheroids yield therapeutic extracellular vesicles that target hypertrophic scar regression via the miR-26a-5p/CCNE2 axis
Source: Burns Trauma. 2025 Jul 22;14:tkaf048. doi: 10.1093/burnst/tkaf048 (PMC13345373; doi:10.1093/burnst/tkaf048)
Supplement: Figure_S2_tkaf048 [file figure_s2_tkaf048.docx]

**Figure S2**


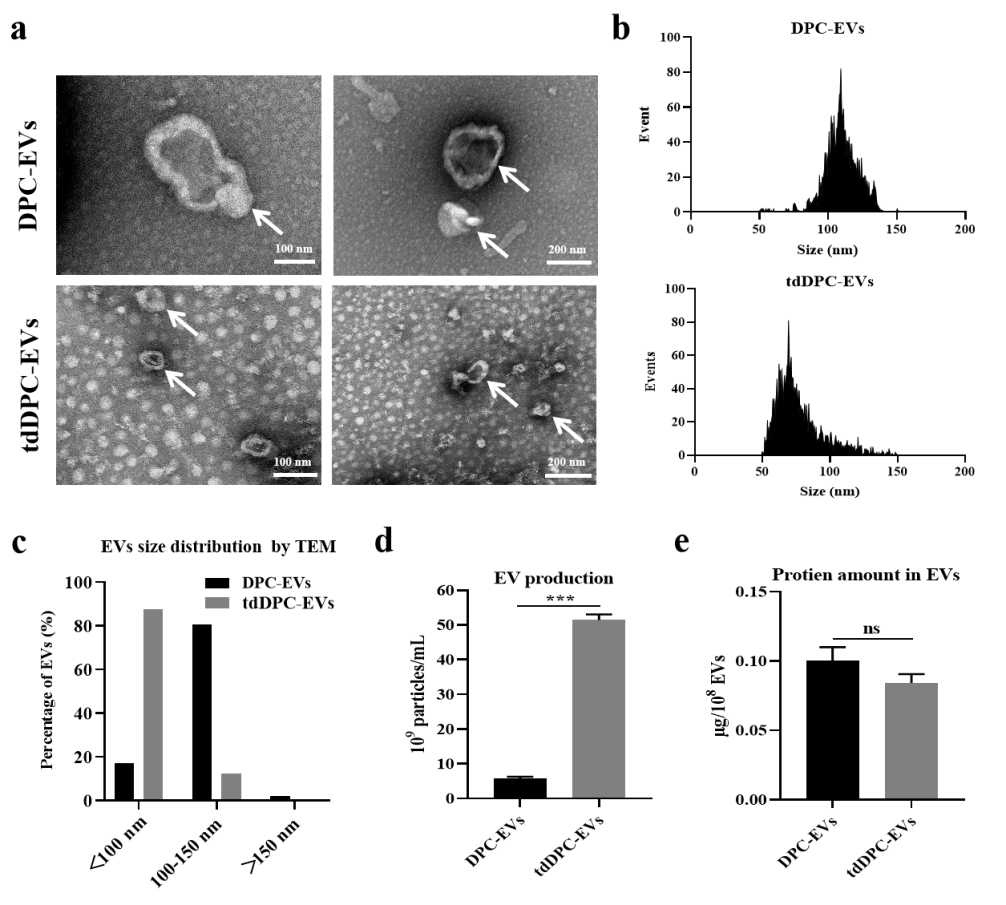


**Figure S2**. Characterization and yield of DPC-EVs and tdDPC-EVs. (a) Ultrastructures of DPC-EVs and tdDPC-EVs visualized via transmission electron microscopy (TEM). Scale bar: 100 or 200 nm. (b) Particle size distributions of DPC-EVs and tdDPC-EVs, as determined using NanoFCM. (c) Distribution determined from the TEM images. (d) EV production from 2D and SFL-3D cultures at 2-day intervals was determined by nanoparticle tracking analysis (NTA) and normalized to the cell number on Day 2 (*n* = 6). (e) Total protein quantification in DPC-EVs and tdDPC-EVs (*n* = 5).
