# Supplementary material for: Self-organizing three-dimensional dermal papilla cell spheroids yield therapeutic extracellular vesicles that target hypertrophic scar regression via the miR-26a-5p/CCNE2 axis
Source: Burns Trauma. 2025 Jul 22;14:tkaf048. doi: 10.1093/burnst/tkaf048 (PMC13345373; doi:10.1093/burnst/tkaf048)
Supplement: Figure_S3_tkaf048 [file figure_s3_tkaf048.docx]

**Figure S3**


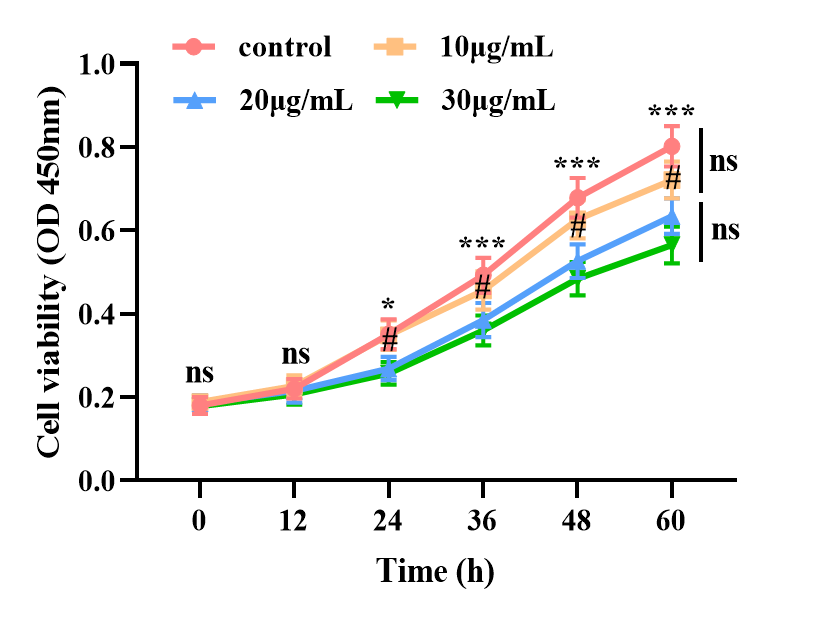


**Figure S3**. Effects of different doses of tdDPC-EV on the viability of HSFs. *n*=4, ns, not statistically significant, **p*< 0.05, ****p* < 0.001 20 μg/ml *vs.* control; #*p* < 0.05 20 μg/ml *vs.* 10 μg/ml.
