# Supplementary material for: Self-organizing three-dimensional dermal papilla cell spheroids yield therapeutic extracellular vesicles that target hypertrophic scar regression via the miR-26a-5p/CCNE2 axis
Source: Burns Trauma. 2025 Jul 22;14:tkaf048. doi: 10.1093/burnst/tkaf048 (PMC13345373; doi:10.1093/burnst/tkaf048)
Supplement: Figure_S4_tkaf048 [file figure_s4_tkaf048.docx]

**Figure S4**

**
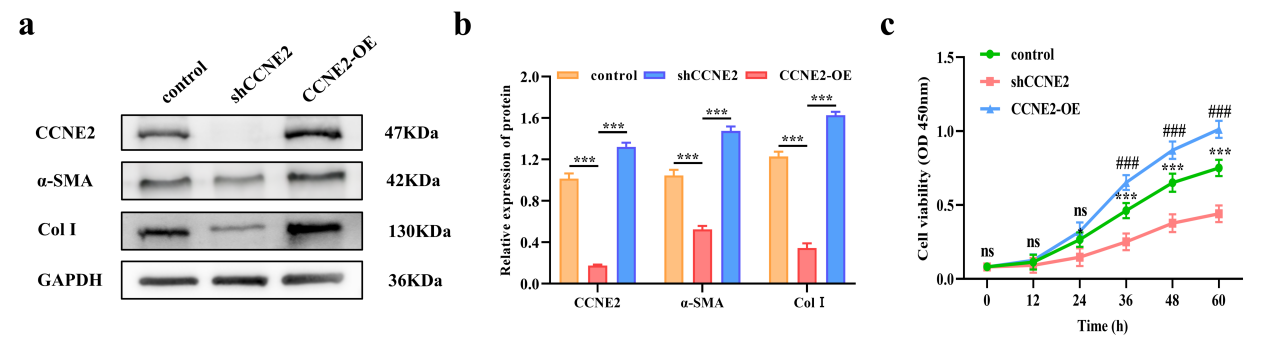
**

**Figure S4**. Knockdown and rescue experiments of CCNE2 in HSFs. (a, b) Western blot analysis showing that the protein expression levels of CCNE2, α-SMA and ColI in HSFs were altered in each treatment group. (c) CCK-8 proliferation assays: CCNE2 knockdown reduces HSF viability, and CCNE2 overexpression increases HSF viability. *n* = 4. (ns, not statistically significant, ****p* < 0.001 shCCNE2 *vs.* control; ###*p* < 0.001 shCCNE2 *vs.* CCNE2-OE).
