# Supplementary material for: Self-organizing three-dimensional dermal papilla cell spheroids yield therapeutic extracellular vesicles that target hypertrophic scar regression via the miR-26a-5p/CCNE2 axis
Source: Burns Trauma. 2025 Jul 22;14:tkaf048. doi: 10.1093/burnst/tkaf048 (PMC13345373; doi:10.1093/burnst/tkaf048)
Supplement: Table_S1_tkaf048 [file table_s1_tkaf048.docx]

| **Table S1.**  **PCR primer sequences for mRNA** | |
| --- | --- |
| **Gene** | **Primer sequences (5’-3’)** |
| CCNE2-forward | GAGCCGACTATGACTACTCAGA |
| CCNE2-reverse | TCACTTTCCGTCTTGTTTTGGG |
| Nanog -forward | GCTTGCCTTGCTTTGAAGCA |
| Nanog -reverse | TTCTTGACTGGGACCTTGTC |
| Sox2-forward | CCCAGCAGACTTCACATGT |
| Sox2-reverse | CCTCCCATTTCCCTCGTTTT |
| Klf4-forward | GATGAACTGACCAGGCACTA |
| Klf4-reverse | GTGGGTCATATCCACTGTCT |
| ColⅠ-forward | CAGTCGATTCACCTACAGCACG |
| ColⅠ-reverse | TTGAAGGAGGATGTTCCCATCT |
| ColⅢ-forward | CCACGGAAACACTGGTGGAC |
| ColⅢ-reverse | GCCAGCTGCACATCAAGGAC |
| α-SMA-forward | TGGCTGATGGAGTACTTC |
| α-SMA-Reverse | GATAGAGAAGCCAGGATG |
| β-actin-forward | GAGCGCGGCTACAGCTT |
| β-actin-reverse | TCCTTAATGTCACGCACGATTT |
| Universal reverse | GTGCAGGGTCCGAGGT |
