# Supplementary material for: Self-organizing three-dimensional dermal papilla cell spheroids yield therapeutic extracellular vesicles that target hypertrophic scar regression via the miR-26a-5p/CCNE2 axis
Source: Burns Trauma. 2025 Jul 22;14:tkaf048. doi: 10.1093/burnst/tkaf048 (PMC13345373; doi:10.1093/burnst/tkaf048)
Supplement: Table_S2_tkaf048 [file table_s2_tkaf048.docx]

| **Table S2.**  **PCR primer sequences for microRNA** | |
| --- | --- |
| **Gene** | **Primer sequences (5’-3’)** |
| miR-182-5p | TTTGGCAATGGTAGAACTCACACCG |
| miR-21a-5p | TAGCTTATCAGACTGATGTTGA |
| miR-148a-3p | TCAGTGCACTACAGAACTTTGT |
| miR-26a-5p | UUCAAGUAAUCCAGGAUAGGCU |
| miR-486a-5p | TCCTGTACTGAGCTGCCCCGAG |
| mimic-nc | UUGUACUACACAAAAGUACUG |
| miR-26a-5p mimic | CCUAUCCUGGAUUACUUGAAU |
| miR-26a-5p inhibitor | AGCCUAUCCUGGAUUACUUGAA |
| U6 | CTCGCTTCGGCAGCACA |
