# Supplementary material for: Self-organizing three-dimensional dermal papilla cell spheroids yield therapeutic extracellular vesicles that target hypertrophic scar regression via the miR-26a-5p/CCNE2 axis
Source: Burns Trauma. 2025 Jul 22;14:tkaf048. doi: 10.1093/burnst/tkaf048 (PMC13345373; doi:10.1093/burnst/tkaf048)
Supplement: supplementary1_tkaf048 [file supplementary1_tkaf048.docx]

**Figure S1**


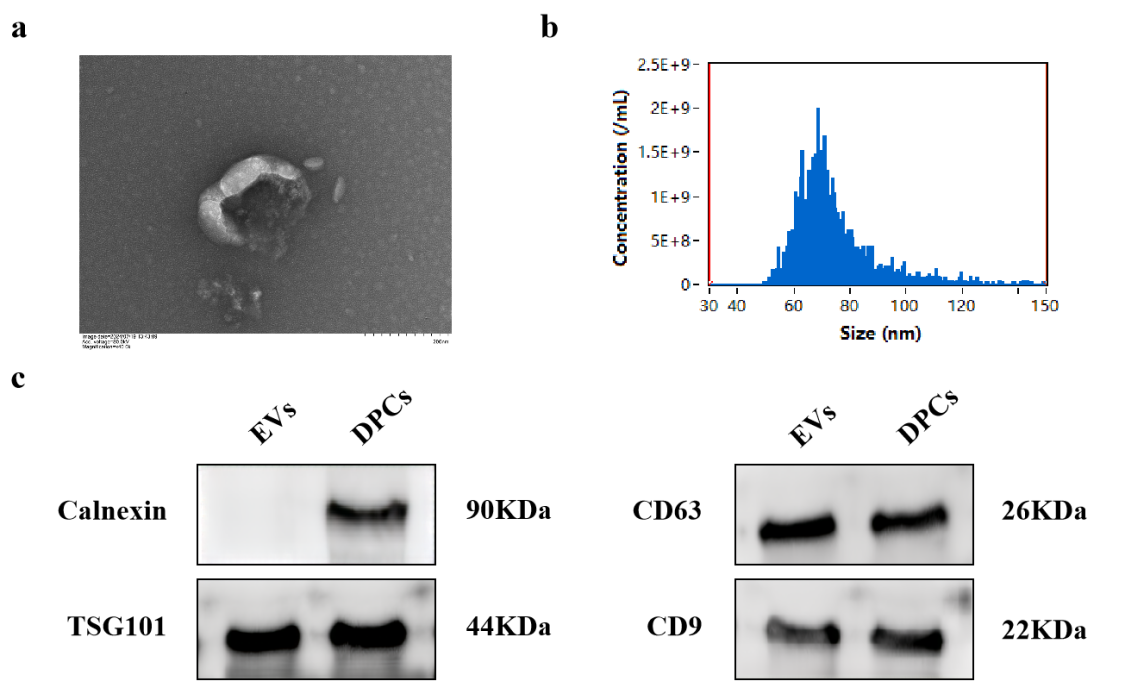


**Figure S1**. Characterization and target cell internalization of 3D dermal papilla cells (tdDPC-EVs). (a) Ultrastructure of tdDPC-EVs visualized via transmission electron microscopy (TEM); scale bar: 200 nm. (b) Particle size distribution of tdDPC-EVs determined using NanoFCM. (c) Analysis of the EV markers TSG101, calnexin, CD81, and CD9 through Western blotting. DPC lysates served as a control. Scale bar: 125 μm.

**Figure S2**


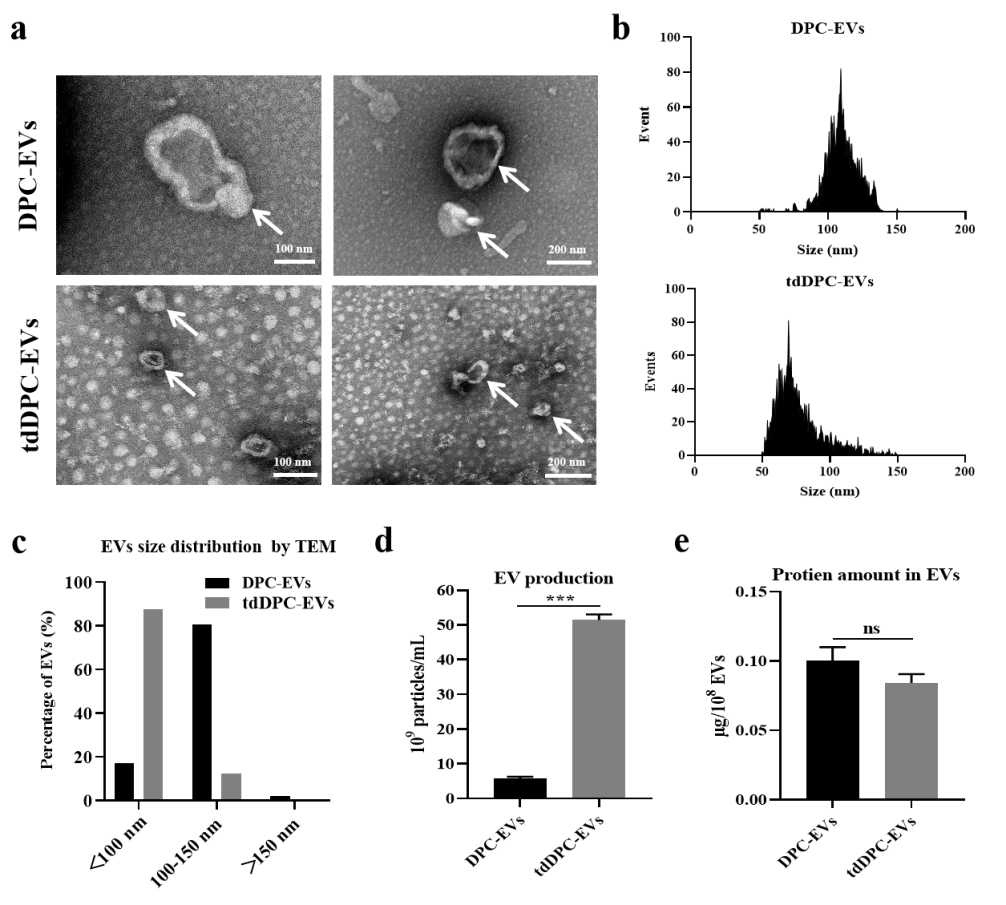


**Figure S2**. Characterization and yield of DPC-EVs and tdDPC-EVs. (a) Ultrastructures of DPC-EVs and tdDPC-EVs visualized via transmission electron microscopy (TEM). Scale bar: 100 or 200 nm. (b) Particle size distributions of DPC-EVs and tdDPC-EVs, as determined using NanoFCM. (c) Distribution determined from the TEM images. (d) EV production from 2D and SFL-3D cultures at 2-day intervals was determined by nanoparticle tracking analysis (NTA) and normalized to the cell number on Day 2 (*n* = 6). (e) Total protein quantification in DPC-EVs and tdDPC-EVs (*n* = 5).

**Figure S3**


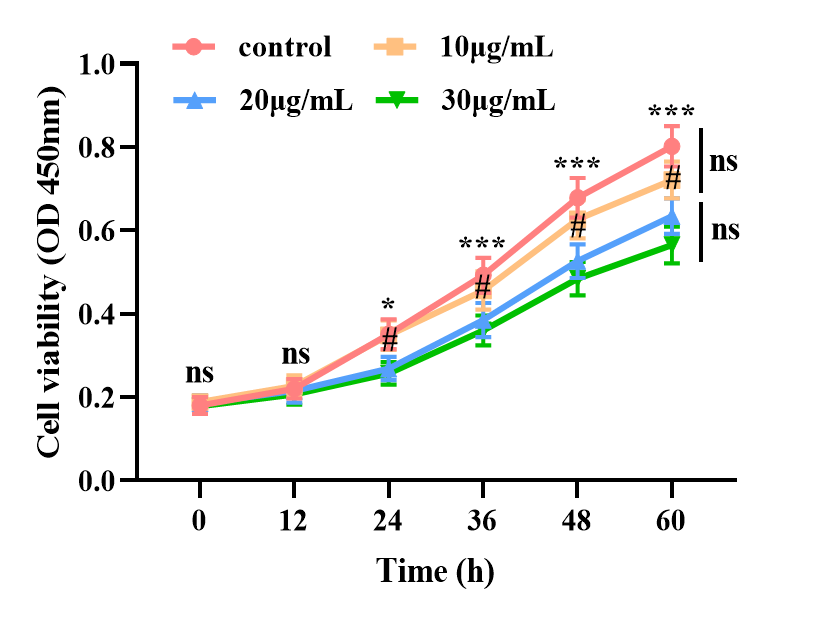


**Figure S3**. Effects of different doses of tdDPC-EV on the viability of HSFs. *n*=4, ns, not statistically significant, **p*< 0.05, ****p* < 0.001 20 μg/mL *vs.* control; #*p* < 0.05 20 μg/mL *vs.* 10 μg/mL.

**Figure S4**


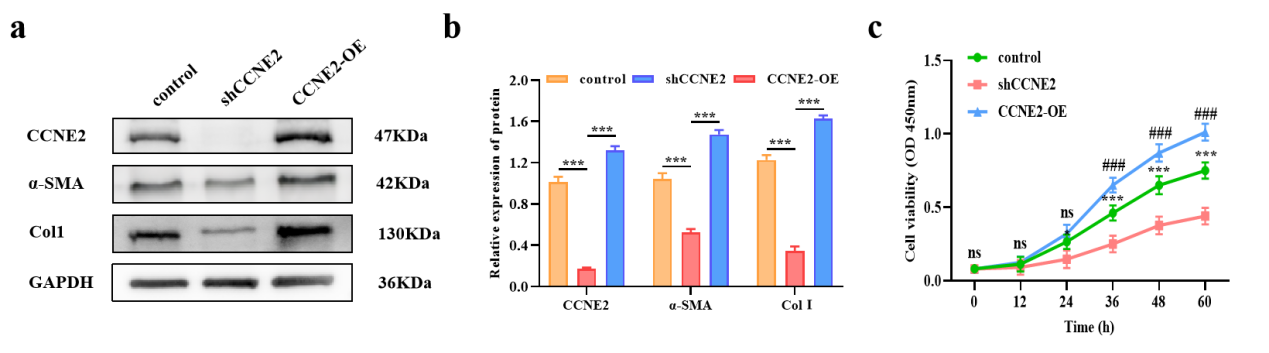


**Figure S4**. Knockdown and rescue experiments of CCNE2 in HSFs. (a, b) Western blot analysis showing that the protein expression levels of CCNE2, α-SMA and ColI in HSFs were altered in each treatment group. (c) CCK-8 proliferation assays: CCNE2 knockdown reduces HSF viability, and CCNE2 overexpression increases HSF viability. *n* = 4. (ns, not statistically significant, ****p* < 0.001 shCCNE2 *vs.* control; ###*p* < 0.001 shCCNE2 *vs.* CCNE2-OE).

| **Table 1.**  **PCR primer sequences for mRNA** | |
| --- | --- |
| **Gene** | **Primer sequences (5’-3’)** |
| CCNE2-forward | GAGCCGACTATGACTACTCAGA |
| CCNE2-reverse | TCACTTTCCGTCTTGTTTTGGG |
| Nanog -forward | GCTTGCCTTGCTTTGAAGCA |
| Nanog -reverse | TTCTTGACTGGGACCTTGTC |
| Sox2-forward | CCCAGCAGACTTCACATGT |
| Sox2-reverse | CCTCCCATTTCCCTCGTTTT |
| Klf4-forward | GATGAACTGACCAGGCACTA |
| Klf4-reverse | GTGGGTCATATCCACTGTCT |
| ColⅠ-forward | CAGTCGATTCACCTACAGCACG |
| ColⅠ-reverse | TTGAAGGAGGATGTTCCCATCT |
| ColⅢ-forward | CCACGGAAACACTGGTGGAC |
| ColⅢ-reverse | GCCAGCTGCACATCAAGGAC |
| α-SMA-forward | TGGCTGATGGAGTACTTC |
| α-SMA-Reverse | GATAGAGAAGCCAGGATG |
| β-actin-forward | GAGCGCGGCTACAGCTT |
| β-actin-reverse | TCCTTAATGTCACGCACGATTT |
| Universal reverse | GTGCAGGGTCCGAGGT |

| **Table 2.**  **PCR primer sequences for microRNA** | |
| --- | --- |
| **Gene** | **Primer sequences (5’-3’)** |
| miR-182-5p | TTTGGCAATGGTAGAACTCACACCG |
| miR-21a-5p | TAGCTTATCAGACTGATGTTGA |
| miR-148a-3p | TCAGTGCACTACAGAACTTTGT |
| miR-26a-5p | UUCAAGUAAUCCAGGAUAGGCU |
| miR-486a-5p | TCCTGTACTGAGCTGCCCCGAG |
| mimic-nc | UUGUACUACACAAAAGUACUG |
| miR-26a-5p mimic | CCUAUCCUGGAUUACUUGAAU |
| miR-26a-5p inhibitor | AGCCUAUCCUGGAUUACUUGAA |
| U6 | CTCGCTTCGGCAGCACA |
